# Integrative Modelling of the Influence of MAPK Network on Cancer Cell Fate Decision

**DOI:** 10.1371/journal.pcbi.1003286

**Published:** 2013-10-24

**Authors:** Luca Grieco, Laurence Calzone, Isabelle Bernard-Pierrot, François Radvanyi, Brigitte Kahn-Perlès, Denis Thieffry

**Affiliations:** 1 Aix-Marseille Université, Marseille, France; 2 TAGC – Inserm U1090, Marseille, France; 3 Institut de Biologie de l'Ecole Normale Supérieure (IBENS), Paris, France; 4 UMR 8197 Centre National de la Recherche Scientifique (CNRS), Paris, France; 5 Inserm 1024, Paris, France; 6 Institut Curie, Paris, France; 7 Inserm U900, Paris, France; 8 Ecole des Mines ParisTech, Paris, France; 9 UMR 144 Centre National de la Recherche Scientifique (CNRS), Paris, France; 10 INRIA Paris-Rocquencourt, Rocquencourt, France; University of Tokyo, Japan

## Abstract

The Mitogen-Activated Protein Kinase (MAPK) network consists of tightly interconnected signalling pathways involved in diverse cellular processes, such as cell cycle, survival, apoptosis and differentiation. Although several studies reported the involvement of these signalling cascades in cancer deregulations, the precise mechanisms underlying their influence on the balance between cell proliferation and cell death (cell fate decision) in pathological circumstances remain elusive. Based on an extensive analysis of published data, we have built a comprehensive and generic reaction map for the MAPK signalling network, using CellDesigner software. In order to explore the MAPK responses to different stimuli and better understand their contributions to cell fate decision, we have considered the most crucial components and interactions and encoded them into a logical model, using the software GINsim. Our logical model analysis particularly focuses on urinary bladder cancer, where MAPK network deregulations have often been associated with specific phenotypes. To cope with the combinatorial explosion of the number of states, we have applied novel algorithms for model reduction and for the compression of state transition graphs, both implemented into the software GINsim. The results of systematic simulations for different signal combinations and network perturbations were found globally coherent with published data. In silico experiments further enabled us to delineate the roles of specific components, cross-talks and regulatory feedbacks in cell fate decision. Finally, tentative proliferative or anti-proliferative mechanisms can be connected with established bladder cancer deregulations, namely Epidermal Growth Factor Receptor (EGFR) over-expression and Fibroblast Growth Factor Receptor 3 (FGFR3) activating mutations.

## Author Summary

Depending on environmental conditions, strongly intertwined cellular signalling pathways are activated, involving activation/inactivation of proteins and genes in response to external and/or internal stimuli. Alterations of some components of these pathways can lead to wrong cell behaviours. For instance, cancer-related deregulations lead to high proliferation of malignant cells enabling sustained tumour growth. Understanding the precise mechanisms underlying these pathways is necessary to delineate efficient therapeutical approaches for each specific tumour type. We particularly focused on the Mitogen-Activated Protein Kinase (MAPK) signalling network, whose involvement in cancer is well established, although the precise conditions leading to its positive or negative influence on cell proliferation are still poorly understood. We tackled this problem by first collecting sparse published biological information into a comprehensive map describing the MAPK network in terms of stylised chemical reactions. This information source was then used to build a dynamical Boolean model recapitulating network responses to characteristic stimuli observed in selected bladder cancers. Systematic model simulations further allowed us to link specific network components and interactions with proliferative/anti-proliferative cell responses.

## Introduction

Mitogen-activated protein kinase (MAPK) cascades can be activated by a wide variety of stimuli, such as growth factors and environmental stresses. They affect diverse cellular activities, including gene expression, cell cycle machinery, apoptosis and differentiation.

A recurrent feature of a MAPK cascade is a central three-tiered core signalling module, consisting of a set of sequentially acting kinases. MAPK kinase kinases (MAPKKKs) are activated following upstream signals. For instance, they can be phosphorylated by small G-proteins belonging to the Ras/Rho family in response to extracellular stimuli. Their activation leads to double phosphorylation and activation of downstream MAPK kinases (MAPKKs), which in turn double phosphorylate MAPKs. Once activated, MAPKs act on their target substrates, which include other kinases and transcription factors [1]. To date, three main cascades have been extensively studied, named after their specific MAPK components: Extracellular Regulated Kinases (ERK), Jun NH2 Terminal Kinases (JNK), and p38 Kinases (p38). These cascades are strongly interconnected, forming a complex molecular network [1]–[4].

MAPK phosphorylation level is regulated by the opposing actions of phosphatases. As the effects of MAPK signalling have been shown to depend on the magnitude and duration of kinase activation, phosphatase action might play an important functional role [5]. Moreover, scaffold proteins bring together the components of a MAPK cascade and protect them from activation by irrelevant stimuli, as well as from negative regulators (such as phosphatases) [6].

The involvement of MAPK cascades in major cellular processes has been widely documented [1], [7], [8]. However, the wide range of stimuli and the large number of processes regulated, coupled with the complexity of the network, raises the fundamental and debated question of how MAPK signalling specificity is achieved [9]. Several interrelated mechanisms have been proposed: opposing action of phosphatases; presence of multiple components with different roles at each level of the cascade (e.g. different isoforms of a protein); interaction with scaffold proteins; distinct sub-cellular localisations of cascade components and/or targets; feedback mechanisms; great variety of molecular signals, as well as distinct durations and strengths; cross-talks among signalling cascades that are activated simultaneously [4], [10]. All these factors contribute to the complexity of the MAPK network and presumably act together to determine signalling specificity.

Deregulations of the MAPK cascades are often observed in cancer [11], [12]. Several components of the network have already been proposed as targets in cancer therapy, such as p38, JNK, ERK, MEK, RAF, RAS, and DUSP1 [12]–[23], but the intricacy of the underlying mechanisms still hinders the conception of effective drugs [24]. A deeper understanding of the regulatory mechanisms involved is crucial to clarify the roles of MAPKs in cancer onset and development, as well as to delineate therapeutic strategies.

During the last decades, mathematical modelling has been widely used to study different aspects of the MAPK cascades [25] (Table S1). Quantitative models (based on Ordinary Differential Equations – ODE) have been proposed to explain the main dynamical properties of the MAPK cascades in relation with their particular structural features (double phosphorylation events, phosphatase effects, feedback loops, role of scaffold proteins, etc.) [26]–[32]. Other modelling studies investigated the behaviour of specific cascades (mainly ERK) leading to MAPK activation in response to external stimuli [33]–[42]. More recently, comprehensive qualitative dynamical models have been developed. Samaga et al. [43] built a large logical model of the signalling network (including MAPKs) responding to Epidermal Growth Factor Receptor (EGFR) stimuli, which was largely derived from the reaction map published by Oda et al. [44]. This model accurately covers the early response of the MAPK cascades to signalling stimuli, with a particular reference to primary and transformed hepatocytes. Also focusing on cancer (in particular, epithelial tumours), the logical model proposed by Poltz and Naumann recapitulates the response of a cell to DNA damaging agents (DNA repair versus apoptotic cell death), and was used to identify candidate target molecules to design novel therapies [45].

In this study, we aimed at better understanding how MAPK signalling deregulations can interfere with tissue homeostasis, leading to imbalance between cell proliferation, on the one hand, and cell growth arrest, possibly followed by apoptotic cell death, on the other hand. The choice between these phenotypes (cell fate decision) is of vital importance in cancer progression: transformed cells receive external and/or autocrine growth stimuli pushing towards cell proliferation (i.e. tumour growth); but they also receive external and/or internal anti-proliferative signals, which coupled with apoptotic stimuli trigger transformed cell clearance from the organism [46]. Our goal was to build a predictive dynamical model able (i) to recapitulate the response of the entire MAPK network to selected stimuli, along with its specific contribution to cell fate decision, and (ii) to assess novel hypotheses about poorly documented mechanisms involved in specific cancer cell types. We focused on urinary bladder cancer, where MAPK network deregulations have been associated with specific phenotypes.

Bladder cancer is the fourth most common cancer among men in Europe and America. Two main types of early stage bladder carcinoma have been delineated so far: (i) non-invasive papillary carcinomas (Ta) are usually mildly aggressive and rarely progress towards higher stages, whereas (ii) carcinomas in situ (Cis) often develop into invasive tumours (T1 to T4 stages) [47]. Activating mutations of Fibroblast Growth Factor Receptor 3 (FGFR3) have been found in 70–75% of Ta tumours, but they are absent in Cis and less frequent (15–20%) in invasive tumours [47], [48]. Oncogenic FGFR3 gene fusions have also been recently identified in a small percentage of invasive bladder tumours [49]. In contrast, over-expression of EGFR has been recurrently associated with higher probability of tumour progression [50]. The mechanisms underlying the cellular response of cancer cells to these signalling stimuli are still poorly understood. Alterations of p53 and retinoblastoma (RB) pathways are presumably involved in tumour progression [51]. These pathways are major controllers of the cell cycle, and the MAPK network presumably regulates their activation by responding to growth factor stimuli. For instance, ERK phosphorylation leads to MYC activation, which can inhibit cell cycle progression through the p14/p53 pathway [52], or activate Cyclin/CDK complexes leading to RB phosphorylation and subsequent E2F-dependent gene transcription [51].

Both EGFR and FGFR3 pathways can activate the MAPK cascades. Although the two signalling pathways largely overlap, the presence of specificity factors might contribute to tune the final cellular response. To tackle these questions, we first compiled published data to build a comprehensive generic reaction map using CellDesigner software [53]–[55]. This map takes into account signals propagating from major stimuli, such as growth factors, cytokines, stress, leading to the activation of MAPKs and their downstream targets, and consequently to cell fate decision. We considered three main cell fates: proliferation, apoptosis, growth arrest.

Next, we used the resulting reaction map to design a qualitative dynamical model with GINsim software [56], [57], which relies on a logical formalism [58]–[60]. To cope with the relatively high number of components, we applied a semi-automatic model reduction procedure to lower the computational cost of dynamical analyses, without losing the main dynamical properties of the system. We then performed logical simulations to check the behaviour of the model in specific documented situations, as well as to predict the behaviour in novel situations. We further investigated the role of positive and negative regulatory circuits in cell fate decision. Altogether, these analyses provided novel insights into the mechanisms differentiating the response of urinary bladder cancer cells to EGFR versus FGFR3 stimuli.

## Methods

### Logical modelling

We built our dynamical model using the logical formalism originally proposed by Thomas [58], [59]. Implemented in GINsim, our logical modelling approach initially requires the delineation of a regulatory graph, where each vertex (node) represents a model component and each arc (signed, directed edge) represents an interaction (activation or inhibition) between two components. Here, all components are associated with Boolean variables, meaning that they can take two possible levels, 0 or 1, denoting the absence/inactivation or the presence/activition of the modelled entities (protein activation level, gene expression level, activation of a cellular process, etc.). The model definition is completed by assigning a logical rule to each component. This logical rule specifies the target value of the component depending on the presence/absence of its regulators, using the classical Boolean operators AND, OR and NOT.

### Logical simulations

The dynamical behaviour of the model can be computed starting from any initial state, step by step, updating the current state according to the logical formulae (logical simulations) [60].

#### Updating policy

Two updating policies are mainly used. According to the synchronous policy, all components are updated simultaneously at each step; consequently, each state has at most one successor. In contrast, according to the asynchronous policy, only one variable can be updated at each step and all the possible successors of a state are computed. Mixed policies based on the notion of priority classes can also be defined using GINsim [61], where subsets of components are ranked. At each step, highest rank variables are then updated in a synchronous or asynchronous way.

In this work, we have used the fully asynchronous updating policy, which usually generates more realistic behaviours [58].

#### State transition graphs and attractors

The dynamics of a logical model can be represented in terms of a state transition graph (STG), in which nodes denote different states of the system (represented by a Boolean vector encompassing the values of all the components), whereas arcs represent enabled transitions between pairs of states. Of particular interest are the states forming attractors, i.e. (groups of) states from which the system cannot escape, which represent potential asymptotical behaviours.

Attractors can be ranged into two main classes:

- stable states, corresponding to fixed points (i.e. states without successors);
- cyclic attractors, corresponding to terminal cycles or to more complex terminal strongly connected components, comprising several intertwined cycles.

Leaning on a representation of the logical rules in terms of Multi-valued Decision Diagrams (MDD), an algorithm enables the computation of all the stable states of a logical model (independently of the initial conditions) [62]. The efficiency of the algorithm (which does not require to compute the state transition graph) makes this tool particularly useful when dealing with large logical models. However, other means are needed to assess the reachability of the stable states from specific initial states, or yet to identify cyclic attractors.

Deeper dynamical analyses imply the computation of the state transition graph. GINsim user can define a set of initial states and an updating strategy; the software then computes the state transition graph, highlighting stable states and cyclic attractors.

GINsim further eases the definition of perturbations, which are simulated by forcing the level of a subset of components to fixed values (or value intervals). For instance, in the Boolean case, we can reproduce a loss-of-function by setting a component to 0, whatever the levels of its regulators, whereas a gain-of-function can be simulated by forcing the corresponding component to 1. More subtle perturbations can be simulated by rewriting relevant logical rules.

As the size of the model considered increases, we are facing the well known problem of the exponential growth of the state transition graph. To cope with this problem, we used two methods that amount to compress the model before simulation or to compress the resulting state transition graph on the fly. These two methods are briefly described hereafter, along with a complementary method enabling the identification of regulatory circuits playing crucial dynamical roles.

### Model reduction

To deal with large models, GINsim enables their reduction by “hiding” selected components [63]. In practice, the user selects the components to hide, and the software hides each of them iteratively, while recomputing the logical rules of their targets. Provided that no functional regulatory circuit is eliminated in the process, this reduction preserves all attractors. For example, the stable states are all conserved, in the sense that each stable state of the reduced model is the projection (on the reduced state space) of a stable state of the original model [63]. This tool is particularly useful when the high dimensionality impedes the computation of the full STG.

### Hierarchical transition graph representation

The analysis of the paths from initial states to attractors can be done directly on the STG when it is small (tens of states), but becomes quickly intractable as the size of STG increases. To cope with this difficulty, we use a novel feature of GINsim, which iteratively clusters the state transition graph into groups of states (components or hyper-nodes) sharing the same set of successors [64]. The resulting hierarchical state transition graph (HTG) displays all the reachable attractors (components at the bottom of the graph), while the other clusters of states represent their basins of attractions (including strict basins with a single outgoing arc targeting a specific attractor, or non-strict basins grouping states that can reach a specific set of HTG components). HTG computation is done on the fly, i.e. without having to store the whole STG, which often leads to strong memory and CPU usage shrinking. Furthermore, this functionality eases the identification of the key commutations (changes of component levels) underlying irreversible choices between the different reachable attractors. Altogether, the HTG representation is very compact (often much more compact than the more classical graph of strongly connected components, as HTG further compacts linear/non circular pathways) and very informative regarding the organisation of the original STG.

### Regulatory circuit analysis

René Thomas proposed generic rules linking the presence of regulatory circuits (simple oriented regulatory cycles) in biological networks with dynamical properties. The first rule states that the existence of a positive circuit (involving an even number of negative regulatory interactions) is a necessary condition for multi-stationarity. The second rule states that the existence of a negative regulatory circuit (involving an odd number of negative regulatory interactions) is a necessary condition for the generation of sustained oscillations [65]. More recently, Remy et al. [66] translated these rules into theorems in the case of asynchronous Boolean dynamical systems (which is the case of our MAPK model).

However, when embedded in a more complex network, specific constraints on the values of the external components acting on circuit components have to be fulfilled in order to allow a regulatory circuit to produce the expected behaviour (“circuit functionality constraints”) [67]. The concept of circuit functionality has been formalised for logical models and implemented into GINsim [62].

GINsim allows to compute all the functional positive and negative circuits of a model. For each of them, the software also provides the corresponding functionality context, defined as a set of constraints on the values of its external regulators. This tool enables the identification of the regulatory circuits playing key dynamical roles within a complex network.

## Results

### MAPK reaction map

Based on published data, we have built and annotated a comprehensive reaction map using CellDesigner (supplementary Dataset S1). This map encompasses 232 species (proteins, genes, complexes, other molecules) and 167 reactions involved in the three most extensively documented MAPK cascades (ERK, JNK, p38). The CellDesigner version of the map (XML format) is provided as supplementary Dataset S2. The MAPK map has been further integrated into the Atlas of Cancer Signalling Networks developed by the group of Emmanuel Barillot at Institut Curie in Paris (https://acsn.curie.fr), where it can be explored using a web browser.

**Figure 1:**
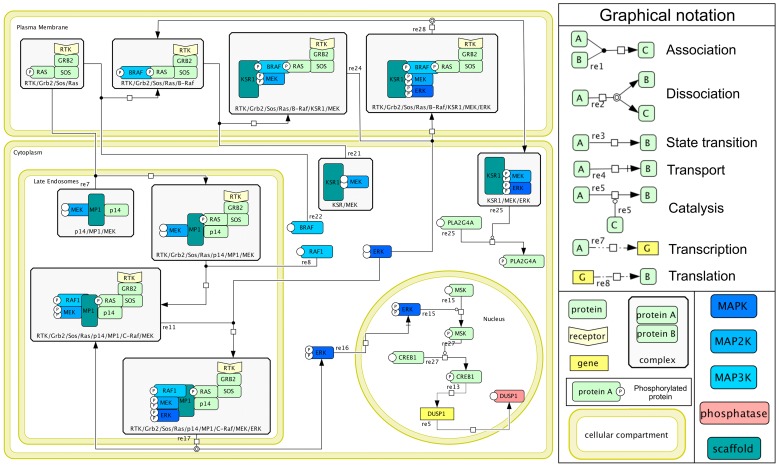
Molecular map for ERK regulation and sub-cellular localisation. After RAS activation, ERK cascade can be recruited and activated on plasma membrane with the help of KRS1 scaffold protein (upper part of the figure); activated ERK is then released (in complex with MEK and KSR1) into the cytoplasm, where it can activate some of its cytoplasmic targets (e.g. PLA2G4A protein). Alternatively, activated receptor complex can translocate to late endosomes (left part of the figure), where ERK cascade can be triggered with the help of MP1 scaffold protein; in this case, activated ERK monomers are released into the cytoplasm, from where they can translocate into the nucleus and exert other effects (e.g. induction of DUSP1 phosphatase). This map is a small fraction of the detailed MAPK network built with the software CellDesigner (www.celldesigner.org) and provided in *png* and *cell* formats (supplementary Dataset S1 and S2).

Our reaction map takes into account signals propagating from different major stimuli, such as growth factors, cytokines, stress, which lead to the activation of MAPKs and their downstream targets. It covers feedbacks and cross-talks among the MAPK cascades, as well as the roles of the best documented phosphatases and scaffold proteins. The main cellular compartments are also represented (plasma membrane, cytoplasm, nucleus, mitochondria, endosomes, etc.), showing the localisation of reactions within the cell. When compartmentalisation has not been fully characterised yet, reactions have been provisionally assigned to the cytoplasm. Proteins are coloured to emphasise relevant families. Figure 1 shows a map excerpt reporting two different mechanisms of ERK activation (see map annotations for more details).

We considered two compartments named “Genes” and “Phenotypes” at the bottom of the map, which include representative genes activated by the MAPK cascades, as well as phenotypes (proliferation, apoptosis, growth arrest) enabled by selected readouts.

We considered information concerning different human and mouse cell types, implying that the MAPK map should be considered as generic. Indeed, at this stage, information is lacking to build a detailed map based exclusively on data for a specific cell type. However, we selected biological events explicitly considered to be cell type independent. When applicable, information concerning cell types is provided through links to relevant database entries (mainly PubMed).

Because the precision of the information retrieved from the literature varies, our map represents different pathways with different levels of details. For instance, we could find detailed information about the scaffold proteins intervening in the ERK cascade and on the sub-cellular localisation of the corresponding protein complexes; in contrast, such information is still largely lacking for the JNK and p38 cascades. This is why the map currently reaches its greatest level of detail for the ERK cascade.

Furthermore, the level of detail represented could also be dictated by readability considerations. For instance, the RTK (receptor tyrosine kinase) component in the map represents several different receptors (e.g. EGFR, FGFR, VEGFR, etc.): all these receptors share common features that are related to MAPK activation. However, their mechanisms of action may differ in some subtle ways, which are not fundamental for our purpose here. The detailed representation of all these pathways would have made the map very difficult to read, and we thus decided to simplify the graphical representation, while keeping track of relevant variations in the annotations of the corresponding species or reactions.

The resulting CellDesigner map constitutes a comprehensive and integrated source of information concerning the roles of the MAPK network in cell fate decision, taking into account specificity factors. This map can be directly used by biologists and modellers to get information about the reported phenomena. It can also be used for visualisation of high-throughput data (e.g. by automatically colouring components based on expression levels) derived from different cell conditions, for example in order to identify differentially expressed components. This can also give insights into cell type-dependent mechanisms.

### MAPK logical model

Hereafter, we focus on the impact of the MAPK network in urinary bladder cancer, with particular emphasis on the differential behaviour between EGFR over-expression and FGFR3 activating mutation.

#### Scope of the MAPK logical model

In order to study the response of the MAPK network to specific stimuli, and its influence on cell fate decision, we built a dynamical model covering the mechanisms reported in the MAPK map. We derived a regulatory graph using the MAPK reaction map as an information source, as detailed in supplementary Text S1. In particular, we considered the following subset of stimuli in our model: EGFR stimulus, FGFR3 stimulus, TGFβR stimulus, and DNA damage. With reference to the latter stimulus, please notice that we did not consider explicitly here the DNA repair process following DNA damage, but we only account for the triggering of growth arrest and apoptosis following sustained DNA damage [68]. In our dynamical model, DNA damage thus corresponds to sustained stress or to the effects of therapies involving DNA-damaging agents.

**Figure 2:**
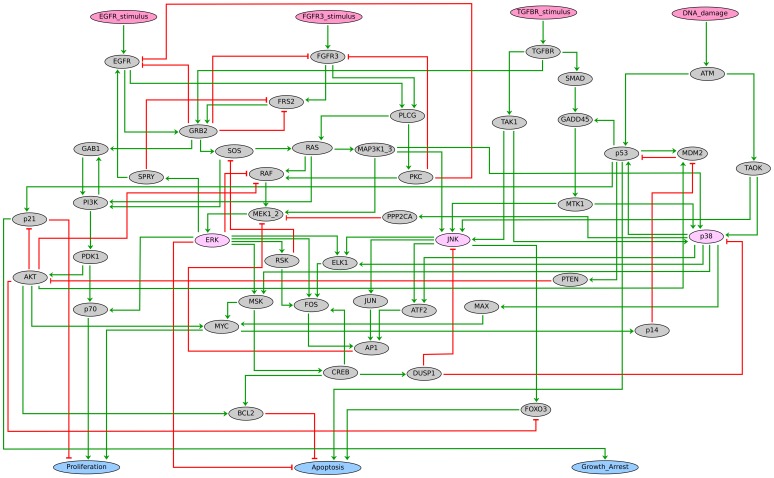
Regulatory graph of the MAPK logical model. Each node denotes a model component. Model inputs, phenotypes and MAPK proteins (ERK, p38, JNK) are denoted in pink, blue and orange, respectively. Green arrows and red T-arrows denote positive and negative regulations, respectively. A comprehensive documentation is provided in the Table S4, which includes a summary of all modelling assumptions, references (PubMed links) and the specification of the logical rule associated with each component. The source file is further provided as supplementary Dataset S4, which can be opened, edited, analysed and simulated with the softare GINsim (www://www.ginsim.org/beta).

The regulatory graph shown in Figure 2 covers the activation of MAPK targets that influence the choice between proliferation, growth arrest, and apoptosis. In particular, we consider MYC and p70 (in the absence of p21) as markers of cell cycle enablement, p21 as a marker of growth arrest, FOXO3 and p53 as markers of apoptosis, whereas ERK and/or BCL2 indicate apoptosis disablement. To ease the interpretation of phenotypes, we defined three output nodes denoting proliferation, growth arrest, and apoptosis, respectively. These nodes represent enablement/disablement of the corresponding processes, depending on the MAPK network state, but not necessarily all requirements for this phenotype. For instance, when proliferation is enabled (by the interplay of MYC, p70 and p21), we assume that Cyclin/CDK complexes are activated. In this context, the node “Proliferation” denotes entry into the cell cycle and does not account for alterations of later stages of the cell cycle. Similar considerations are applicable for the other phenotypes modelled. This simplification is justified by our focus on the specific contributions of the MAPK network to cell fate decision.

Each of the 52 components of the regulatory graph is modelled by a Boolean variable along with a logical rule (Table S2) specifying how the component activity depends on its regulators.

#### Reduced model versions

To cope with the relatively high dimension (52 components) of our model, we took advantage of the model reduction function implemented in GINsim (see Methods). Indeed, it is difficult to perform simulations with the original model version, as it entails 2^52^ states. The choice of components to hide depends on the simulation performed. For instance, if we plan to simulate a p53 loss-of-function and observe its effects on p21, we better conserve p53 (otherwise we cannot define the perturbed version) and p21 (otherwise, we cannot explicitly observe its value). However, as we wanted to test several situations, almost half of the MAPK model components were needed to be observable. Consequently, we designed several reduced versions of the original MAPK model, each of which dedicated to a subset of simulations. This strategy enabled us to drastically reduce the computational cost of our in silico experiments (the dimension of the reduced models ranged from 16 to 18 components).

**Figure 3:**
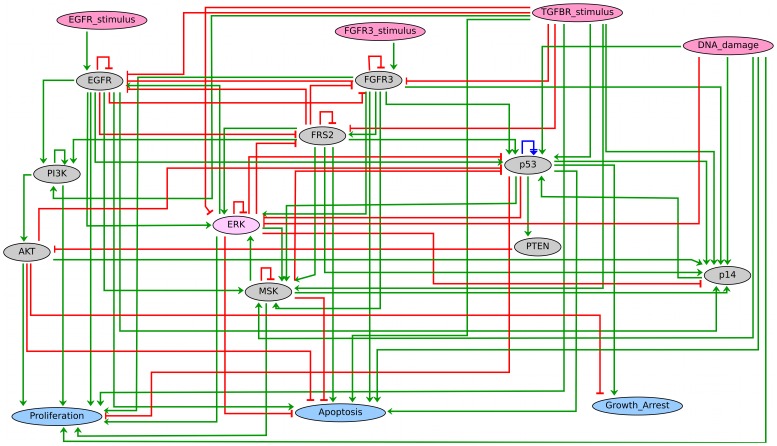
Regulatory graph of a reduced version of the MAPK model. The regulatory graph corresponds to the “red1” reduced model version (cf. supplementary Table S3, column 1). To obtain this version, the preservation of pink and blue nodes was imposed, along with that of {EGFR, FGFR3, p53, p14, PI3K, AKT, PTEN, ERK}, in order to investigate the effects of perturbations affecting these components. The remaining nodes {FRS2, MSK} were maintained by the reduction algorithm because of the occurrence of auto-regulatory loops during the reduction process. Green arrows and red T-arrows denote positive and negative regulations, respectively, whereas blue arrows denote dual interactions.

Altogether, we defined three different reduced model versions, whose component lists are reported in supplementary Table S3. These definitions are enclosed in the comprehensive model file (supplementary Dataset S4) and enable the generation of reduced versions according to simulation needs. Figure 3 shows the regulatory graph corresponding to one reduced version, namely, “red1”. Supplementary Dataset S3 lists the simulations performed for each reduced version. The three model reductions were found equivalent in terms of attractors and main dynamical properties. Briefly, reduction “red1” was used to obtain the results discussed in the sections “Coherence with well established bladder cancer deregulations” and “Predictions generated with the MAPK logical model”, while reductions “red2” and “red3” were used to obtain the results discussed in the section “Coherence with additional cancer-related facts”.

### Coherence of the logical model behaviour with published data

The logical rules assigned to model components were inferred from information about a broad range of experiments and cellular conditions. To check the coherence of the global behaviour of the resulting model with current biological knowledge, we systematically compared its dynamical properties with published data concerning different tumoural cell types, with particular attention to bladder cancer. More specifically, we first assessed the dynamical behaviour of the model under well established perturbations observed in the bladder cancer subtypes of interest. We further checked the coherence of the model behaviour with an additional list of biological facts, not necessarily involved in bladder cancer. These analyses were carried out by performing asynchronous simulations for selected initial conditions (initial states, input signals, potentially in the presence of perturbations), and observing the attractors reached by the system.

In practice, the entire process from reaction map construction to model simulations is iterative, requiring several rounds of literature searches and *in silico* experiments. Whenever the model disagreed with established facts, we went back to the literature to seek complementary information and refined our modelling hypotheses. The reaction map and the logical model where systematically and consistently completed with relevant information during this process.

#### Coherence with well established bladder cancer deregulations

As we build our model around the comparison between EGFR over-expression and FGFR3 activating mutation in bladder cancer, our first simulations were dedicated to the assessment of the model behaviour in these circumstances.

**Figure 4:**
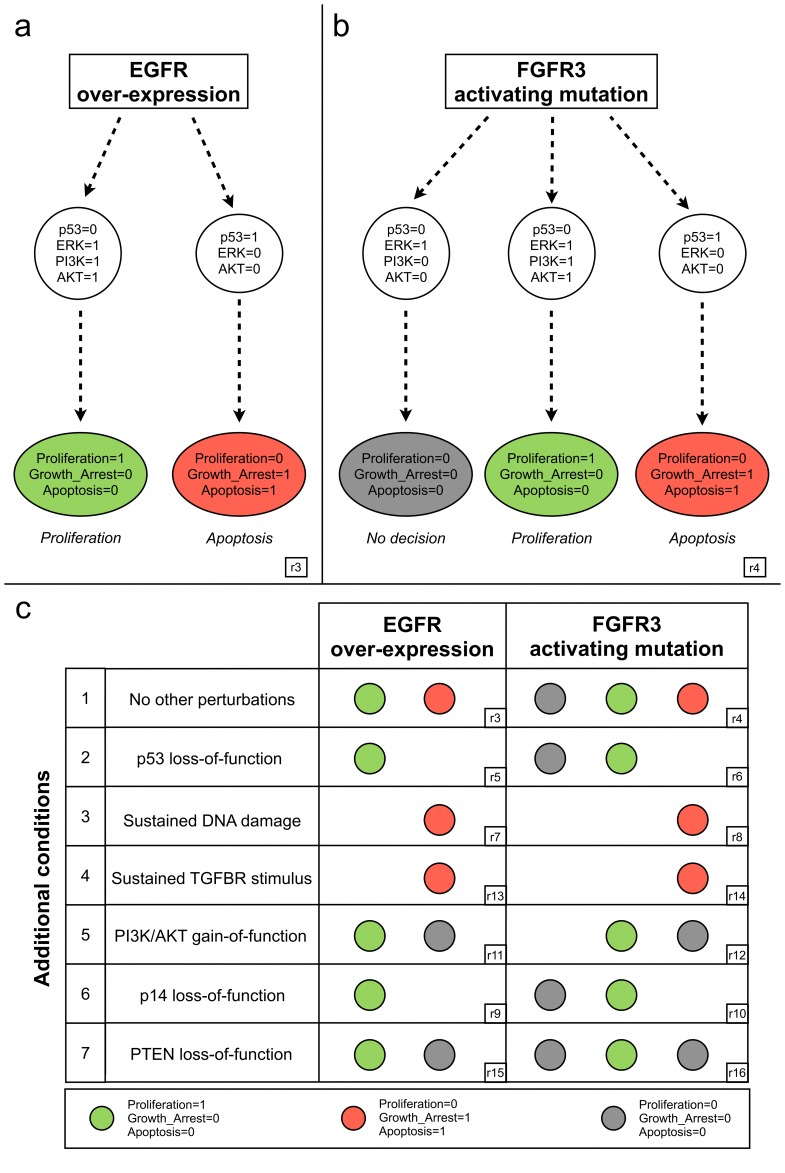
Coherence of the logical model with well established bladder cancer deregulations. **a**) Simplified representation of the model dynamics following EGFR over-expression (EGFR = 1 throughout the simulation and all inputs set to 0 throughout the simulation). If p53 is activated first (right branch), an apoptotic attractor is reached, characterised by inactivation of ERK and AKT. If ERK and PI3K are activated first (left branch), then p53 is inactivated and AKT is activated, leading to a proliferative attractor. **b**) Simplified representation of the model dynamics following FGFR3 activating mutation (FGFR3 = 1 and all inputs set to 0 throughout the simulation). When p53 is activated first (right branch), an apoptotic attractor is reached, characterised by inactivation of ERK and AKT. If ERK is activated first (left and central branches), then p53 is inactivated. When PI3K is also activated (central), a proliferative attractor is reached, characterised by activated AKT. In contrast, when PI3K is not activated (left), the cell fails to make a clear decision at the level of the MAPK network. **c**) Attractors reached by the model in presence of receptor alterations, coupled with additional common deregulations observed in bladder cancer. Coloured circles denote the phenotypes characterising the attractors reached in each situation (we used the same colour code as in panels a and b, while empty spaces denote the loss of the corresponding branch in the state transition graph). Identifiers in rectangles (e.g.. r3, r4, etc.) point to simulation results reported in more details in Dataset S3 and Text S2.

Figure 4a reports a simplified view of the model dynamics following EGFR over-expression, obtained by setting EGFR to 1 throughout the simulation, in the absence of additional stimuli (see supplementary Dataset S3 for precise simulation settings). In response to EGFR over-expression, the asynchronous state transition graph encompasses two sets of trajectories: one characterised by p53 activation and ERK silencing, leading to an apoptotic stable state (i.e. Apoptosis = Growth_Arrest = 1, Proliferation = 0); the other characterised by p53 silencing and ERK activation, leading to a PI3K/AKT-dependent proliferation stable state (i.e. Apoptosis = Growth_Arrest = 0, Proliferation = 1) (cf. simulation provided in the supplementary Text S2).

Alterations in the p53 pathway have been associated with more aggressive and invasive bladder cancers [50]. The fundamental role of p53 in the model can be observed by simulating a p53 loss-of-function (second row of Figure 4c). In this case, the proliferative attractor is kept, while the apoptotic attractor is lost. In contrast, still in presence of EGFR over-expression, when the system is also subjected to persistent DNA damage (third row of Figure 4c), we obtain a single apoptotic attractor. This behaviour is in agreement with the fact that when damage is moderate (i.e. absence of DNA damage stimulus), the cell is still able to escape apoptotic cell death by down-regulating p53 signalling (i.e. possible switch between the two attractors of Figure 4a), but p53 eventually induces apoptosis in cells subjected to extensive DNA damage [69].

A similar response is also predicted in the case of TGFBR stimulus, in line with the typical anti-proliferative role played by this pathway [70]. In this respect, TGFBR has also been shown to induce proliferation in tumours, in some circumstances, but the conditions (especially in relation with MAPK network) under which this occurs are still poorly understood [70]. Strong activation of PI3K/AKT pathway has also been associated with enhanced bladder tumour proliferation [50]. Accordingly, in our model, PI3K/AKT gain-of-functions counteract p53 pathway effects (fifth row of Figure 4c), impairing the apoptotic phenotype.

Two other important loss-of-function known to be associated with poorer prognosis in bladder cancers have been simulated. Deletions of either PTEN or p14 in our model are associated with a more aggressive phenotype (sixth and seventh row of Figure 4c). The former is a tumour suppressor shown to inhibit AKT expression [71]. The latter is induced by MYC and is able to enhance p53 activity by inhibiting MDM2 [51]. According to our model, p14 loss-of-function abrogates apoptosis, which accounts for the observed dual role of MYC [72]: on the one hand, MYC contributes to proliferation (MYC is a read-out for proliferation in the model); on the other hand, it is involved in p53-dependent apoptosis.

The behaviour of our model in the case of FGFR3 activating mutation is depicted in Figure 4b (cf. Text S2 for a complete simulation). We find again the “p53 versus ERK” pattern accounting for the fundamental role of p53 in cell fate decision. Interestingly, the non-apoptotic branch of the asynchronous state transition graph is now characterised by two different behaviours. On the one hand, similarly to EGFR over-expression, when PI3K is active, the system will eventually reach a proliferative attractor. On the other hand, a p53-independent PI3K/AKT pathway leads to an attractor characterised by all phenotypes set to 0, that we interpret as “no cell fate decision taken” at the level of MAPK network. This is coherent with the contention that FGFR3 mutations, mainly characterising non-invasive bladder carcinomas, relatively mildly induce proliferation due to the action of ERK cascade [47], [51], [73]. Indeed, the coexistence of these outcomes (proliferation versus no cell fate decision) tentatively explain the less aggressive phenotype generally observed in FGFR3-mutated bladder carcinomas, in comparison with urothelial tumours over-expressing EGFR (associated only with a proliferative attractor). The underlying mechanisms are further analysed below. By and large, the simulations of FGFR3 mutation correctly recapitulate the effects of the major bladder cancer deregulations listed in Figure 4c (left column), producing results that are qualitatively coherent with those described for EGFR over-expression.

#### Coherence with additional cancer-related facts

To further assess the consistency of the behaviour of our model with current knowledge, we selected a list of established facts regarding the effects of perturbations on (human/mouse) cancer cells, not necessarily bladder-related. Based on this list, we defined a series of in silico experiments, combining relevant initial states and virtual perturbations (loss-of-functions and/or gain-of-functions of selected model components, as defined in Methods).

This analysis mainly consisted in cross-checking the attractors obtained in our simulations with the qualitative information retrieved from the literature, without focusing on the full dynamics of the system.

A summary of the results obtained is provided in Table 1 (for more details, see the supplementary Dataset S3). Additionally, all the simulations performed can be easily reproduced using the model files available as supplementary Dataset S4. Briefly, the involvement of MAPKs in cell fate decision was assessed through perturbations of relevant components. The model accounts for the pro-apoptotic role of p38 and JNK, as well as for the promotion of growth arrest by p38, and for the proliferative role of ERK. The model also recapitulates the p21-mediated tumour suppressor function of p53, along with the impairment of this function due to epigenetic silencing of GADD45. Additionally, we were also able to reproduce (i) the positive effects of EGFR/FGFR3/RAS/RAF over-expressions on ERK activation; (ii) the negative effects of HSP90-inhibitors on cell proliferation; (iii) the role of ERK against anti-proliferative TGFβ signalling; and (iv) the role of JNK against RAS-induced proliferation. These simulations cover the most salient behaviours of the model, showing a remarkable coherence with published data.

**Table 1:** Coherence of model simulations with published experimental evidence.

Reduction	Simulation	Biological data	Model behaviour
red2	r17, r18	* RAF or RAS over-expressions can lead to constitutive activation of ERK. [11]	In absence of inputs, constitutive activity of any one among RAF or RAS can lead to permanent ERK activation, associated with proliferation.
red2	r19	* HSP90-inhibitor disrupts RAF, AKT and EGFR, leading to successful cancer treatment [86].	Concomitant RAF, AKT, EGFR deletions abrogate the proliferative stable states observed in the unperturbed model, both in the case of EGFR over-expression (obvious – simulation not performed) and in the case of FGFR3 activating mutation.
red2	r20, r21, r22, r23	* Patients with p53-altered/p21-negative tumors demonstrated a higher rate of recurrence and worse survival compared with those with p53-altered/p21-positive tumors [87].	Following either EGFR over-expression or FGFR3 activating mutation, concomitant p21 and p53 loss-of-functions correspond to a phenotype characterised by apoptosis escape (Apoptosis = Growth_Arrest = 0), with the possibility to attain proliferation. Association of p53 loss-of-function and p21 gain-of-function leads to growth arrest attractors, all characterised by no proliferation.
red3	r25	p38 and JNK play important roles in stress responses, such as cell cycle arrest and apoptosis [7], [69].	In presence of either DNA_damage or TGFBR_stimulus, growth arrest/apoptosis stable states are all lost in the p38/JNK-deleted model.
red3	r26	p38 and JNK, especially in the absence of mitogenic stimuli, have been shown to induce apoptotic cell death [7], [69].	When p38/JNK are constitutively active, apoptotic attractors (Growth_Arrest = Apoptosis = 1, Proliferation = 0) are obtained in the absence of other stimuli.
red3	r27	p38 plays its tumour suppressive role by promoting apoptosis and inhibiting cell cycle progression [11].	Under JNK constitutive activation, p38 loss-of-function determines loss of apoptotic attractors obtained in r26.
red3	r28	JNK may contribute to the apoptotic elimination of transformed cells by promoting apoptosis [11], [88].	Under p38 constitutive activation and JNK loss-of-function, all apoptotic attractors obtained in r26 become growth arrest attractors (Growth_Arrest = 1, Apoptosis = 0, Proliferation = 0), thus determining loss of apoptotic attractors obtained in r26.
red3	r29	Epigenetic gene silencing of GADD45 family members has been frequently observed in several types of human cancers [69].	In presence of DNA_damage (main GADD45 activator), Growth_Arrest and Apoptosis components permanently oscillate when GADD45 is silenced, suggesting less propensity to cell death. Apoptotic stable states are still reached in presence of TGFBR_stimulus
red3	r30	ERK increases transcription of the cyclin genes and facilitates the formation of active Cyc/CDK complexes, leading to cell proliferation [89].	ERK gain-of-function always leads to proliferative attractors (Proliferation = 1, Growth_Arrest = Apoptosis = 0), in the absence of other stimuli.
red3	r31	ERK disrupts the anti-proliferative effects of TGFβ [11].	Whereas TGFBR_stimulus leads to an apoptotic stable state (r24), coupling of TGFBR_stimulus with ERK gain-of-function only leads to permanent growth arrest (Growth_Arrest = 1, Apoptosis = 0).
red3	r32	JNK might reduce RAS-dependent tumour formation by inhibiting proliferation and promoting apoptosis [88].	In absence of other stimuli, JNK constitutive activation completely abrogates RAS-dependent proliferation following RAS over-expression (r18). Instead, apoptotic attractors are always reached.

Asterisks denote facts explicitly related to bladder cancer, whereas unmarked entries correspond to generic or loosely specified mechanisms. Full simulation results can be found in Dataset S3, with the help of the identifiers provided in the first two columns.

### Predictions generated with the MAPK logical model

Having shown that our MAPK model is consistent with published data, we designed additional simulations to explore novel mechanistic hypotheses.

#### ERK-related feedback mechanisms

So far, we have described the behaviour of our model in presence of tumoural deregulations of growth factor receptors. Let us consider now what happens when the expression of such receptors is not altered (i.e. unperturbed logical rules for either EGFR or FGFR3 variables), in presence of sustained growth factor stimulation (i.e. either EGFR_stimulus or FGFR3_stimulus set to 1 throughout the simulations). The attractor reached from normal EGFR stimulation is characterised by oscillations (between 0 and 1) of the values of EGFR, ERK and p53, thus leading to oscillations of phenotype variables, in particular for “Proliferation” (Dataset S3 – r1). This behaviour contrasts with the well defined phenotypes obtained following EGFR over-expression (Figure 4a). It can be interpreted as the impossibility to obtain sustained activation (or inactivation) of the considered actors in presence of sustained growth factor stimuli. In other words, ERK oscillations in our state transition graph correspond to the transient ERK activation in the ODE-based model from Orton et al. [38]. Similar results are obtained for FGFR3 stimulation (Figure 4b and Dataset S3 – r2), in agreement with literature [74].

The main negative feedbacks underlying such responses are acting directly on the receptors (Figure 2): one accounting for GRB2-dependent ubiquitination and degradation of the receptors [75]; the other accounting for PKC-mediated negative effects [74], [76]. Concomitant disruptions of these feedbacks in our model lead to simulation results equivalent to those obtained with receptor gain-of-function (cf. Figure 2 and Table S2), whereas disruption of any other downstream negative feedback does not qualitatively influence the outcome (data not shown). This is also in agreement with the results obtained by Orton et al., who proposed that the degradation of receptors (e.g. rather than SOS inhibition by RSK) could be the main mechanism determining a transient activation of ERK pathway in response to growth factors.

#### Role of Sprouty-mediated feedbacks

According to our model simulations (Figure 4a), when EGFR is over-expressed (e.g. in the presence of an autocrine signal), in the absence of p53 activation, the outcome is proliferation. In contrast, when FGFR3 stimulus is present, two possible outcomes are observed in the absence of p53: a proliferative stable state and a non-proliferative stable state, the later with all phenotype variables set to 0.

General hypotheses involving the interplay between the p53, RB and ERK pathways have been proposed to explain the different phenotypes experimentally observed in bladder carcinomas [50], [73], but the precise mechanisms have not been elucidated yet. A closer analysis of the regulatory graph shown in Figure 2 reveals several feedbacks. Interestingly, ERK exerts a positive feedback on EGFR but a negative feedback on FGFR3-activated FRS2, through Sprouty (SPRY in the model). Intuitively, this suggests that ERK strengthens EGFR stimulus but weakens FGFR3 stimulus, potentially explaining the different phenotypes observed. Additionally, GRB2 exerts a negative feedback on FRS2, which is in turn specifically activated by FGFR3.

**Figure 5:**
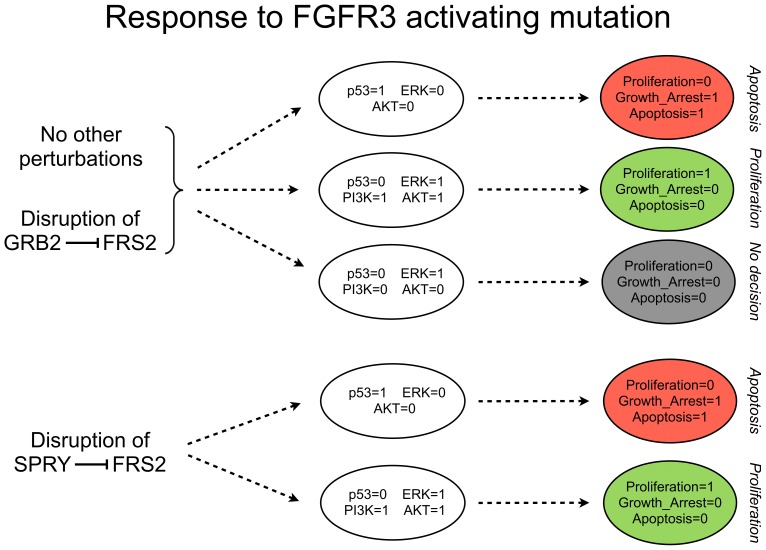
FGFR3 activating mutation and role of SPRY. Simulations were performed under FGFR3 gain-of-function (FGFR3 = 1 and all inputs set to 0, throughout the simulations). Simplified model dynamics are shown as in Figure 4a–b. Results are shown for the wild type model (red1 model reduction), as well as for perturbed model versions obtained by disrupting the inhibition of interest.

Disruption of EGFR activation by SPRY does not play an important role in the case of EGFR over-expression (which indeed corresponds to setting EGFR to 1 independently of its regulators). FRS2 inhibition by SPRY, but not by GRB2, tentatively plays an important role in the response of the MAPK network to FGFR3 activating mutation. Indeed, disrupting the latter inhibition (Figure 5) does not affect significantly the model behaviour. On the contrary, comparison of Figures 4a–b and Figure 5 indicates that SPRY-dependent inhibition of FRS2 might be the key to explain the difference between the responses to EGFR and FGFR3 stimulations (i.e. in the absence of this inhibition, the model behaves equivalently in the two cases).

To further clarify these mechanisms, we considered the role of functional positive circuits, which are known to promote multi-stable behaviours (cf. Methods). According to our model analysis, the GAB1-PI3K-GAB1 circuit underlies the coexistence of the two stable states found in the presence of FGFR3 activating mutation, but in the absence of p53 activation. We thus propose that this circuit plays a fundamental role in FGFR3 signalling, constituting a switch between proliferative and non-proliferative phenotypes. The underlying mechanisms can be further clarified by a careful analysis of Figure 2. On the one hand, following FGFR3 stimulus, when PI3K activation is faster/stronger than ERK activation, cell proliferation is enabled, because PI3K is definitively activated (due to the action of GAB1-PI3K-GAB1 positive circuit) and can then inhibit p21 through the PDK1/AKT pathway. ERK is then also activated, enhancing proliferation together with PI3K. On the other hand, upon ERK activation (coming from a rapid GRB2 and/or PKC mediated signalling), if the inhibition of GRB2 through the SPRY/FRS2 feedback is faster/stronger than PI3K activation, then PI3K cannot be activated anymore. In this scenario, ERK would rather contribute to disable cell proliferation.

Our model thus predicts that the strength/rapidity of PI3K activation versus SPRY-mediated ERK negative feedback could underly the less aggressive phenotypes observed in FGFR3-mutated bladder carcinomas.

#### MAPK cross-talk mechanisms

Using our logical model, we further addressed the roles of cross-talks between the different MAPK cascades, in particular those involving phosphatases.

**Figure 6:**
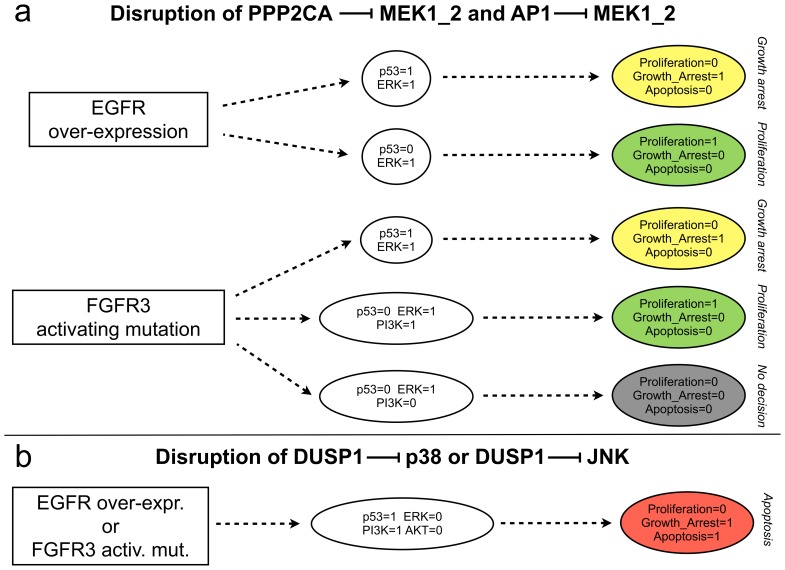
Analysis of MAPK cross-talks by disruptions of specific interactions. **a**) Effects of the disruptions of the inhibitions of MEK by AP1 and the phosphatase PPP2CA. **b**) Effects of the disruption of the inhibition of p38 or of JNK by the phosphatase DUSP1. These simulations were performed after removing the corresponding interactions and blocking the level of the perturbed receptor to level 1 (with all inputs set to 0, throughout the simulation).

We first analysed the negative cross-talks from p38/JNK cascades towards ERK cascade, which involve MEK inhibition by AP1 and the phosphatase PPP2CA [2]. In the context of either EGFR over-expression or FGFR3 activating mutation (Figure 6a), the disruption of these inhibitions mainly lead to the loss of apoptotic attractors (compare Figure 6a with Figure 4a–b). The lost attractors are “replaced” by new attractors characterised by growth arrest alone. These two cross-talks are thus presumably important for the triggering of apoptotic responses in the presence of growth factor receptor alterations. Indeed, in the absence of such cross-talks, p53 pathway is only able to induce growth arrest, but not apoptosis, precluding a complete anti-proliferative response. This is also true in the concomitant presence of DNA damage stimulus (data not shown).

Finally, we examined the roles of p38 and JNK inhibitions by DUSP1 [77]. Following receptor (either EGFR or FGFR3) alteration, disruption of any of these two inhibitions results in a persistent silencing of ERK and a persistent activation of p53, as well as an activation of PI3K and an inactivation of AKT, which ultimately lead the system towards an apoptotic attractor (compare Figure 6b with Figure 4a–b). DUSP1-mediated cross-talks between the MAPK cascades thus tentatively underly proliferative responses in presence of growth factor receptor alterations, presumably via the inhibition of p53 pathway.

## Discussion

We have presented a bottom-up modelling approach to gain insights into the influence of the complex MAPK signalling network on cancer cell fate decision. We started by collecting pieces of information from the literature and assembling them into a detailed reaction map, which served as source of information for further dynamical modelling. The resulting map is generic, meaning that it was drawn by using information coming from different experimental models.

Based on specific biological questions, our dynamical logical modelling involved the abstraction of relevant information from the map and the drawing of a qualitative influence network (regulatory graph). Next, we assigned consistent logical rules to each component to enable logical simulations. In order to perform detailed analyses at reasonable computational costs, we derived reduced model versions preserving the main dynamical properties of the original model. The reduced versions can be considered as further abstractions of the MAPK network, explaining its qualitative behaviour in terms of selected molecular actors. Despite the fact that we made no use of quantitative data, and that we finally represented an extremely complex signalling network through a limited number of Boolean components, we were able to recapitulate its behaviour for diverse documented biological conditions. These results set the background to investigate the roles of poorly documented regulatory mechanisms.

In this modelling study, we particularly focused on bladder cancer. Importantly, our simulations qualitatively recapitulated salient phenotypic differences observed in invasive *versus* non-invasive carcinomas, and allowed us to formulate reasonable hypotheses concerning the mechanisms determining such differences. These hypotheses are readily amenable to experimental validation.

Our MAPK network model can be considered as a module for the assembly of more comprehensive cancer-related network. From this point of view, it will be interesting to merge our model with other logical models implementing other aspects of cell fate decision, in particular the model proposed by Calzone et al. [78], which covers the interplays between NFκB pro-survival pathway, RIP1-dependent necrosis, and extrinsic/intrinsic apoptosis pathways.

In the Introduction, we highlighted the importance of specificity factors in determining signal specificity of the MAPK network and took this into consideration in the construction of the reaction map. However, given the heterogeneity of available information among the different MAPK cascades, we could not include all these factors in our logical model. Nonetheless, we considered some of them, including several feedbacks, cross-talks, phosphatases and input stimuli. These allowed us to focus on interesting aspects and identify mechanisms potentially underlying the different responses of bladder cancer cells to different growth factor receptors (EGFR versus FGFR3).

The role of SPRY-dependent down-regulation of FGFR3 signalling seems to be determinant for the decision between proliferative and non-proliferative response. Moreover, the model also suggests that the presence of PI3K/AKT, but not ERK, positively correlates with the presence of a proliferative phenotype. Nevertheless, ERK-related mechanisms (fastness/strength of ERK activation and activation of SPRY) seem to be determinant for driving the switch.

Such different responses provide a striking example of how signals transduced by largely overlapping pathways can produce opposite effects. To explain this behaviour, we analysed the roles of specific model circuits, which are presumably extremely important in the phenotype choice. Our data further highlight the contribution of cross-talks among the MAPK cascades to cell fate decision. Other specificity factors, including scaffold proteins and sub-cellular localisation, should also be taken into consideration in the near future, as novel data on these factors will be gathered. This will require a regular updating of our MAPK reaction map, by including new findings related to cell fate decision.

We interpreted the p53-independent response of the MAPK network to FGFR3 stimulus as a sort of balance between proliferative and non-proliferative phenotype. A decreased rate of cell proliferation might indeed explain the less aggressive phenotypes frequently observed in FGFR3-mutated bladder carcinomas, in comparison with EGFR over-expression cases. Interestingly, this behaviour can be further related with opposite effects of FGFR3 activation in other cell types. In particular, activating FGFR3 mutations have been associated with growth arrest in chondrocytes, whereas they enhance proliferation and/or transformation in several cancer types and skin disorders (e.g. bladder cancer, multiple myeloma, seborrheic keratosis, etc.) [79]. Tentatively, proper modifications (e.g. concerning the introduction of STAT-dependent pathways and tuning of AKT response to growth factors [80]) may enable our MAPK model to account for these observations.

Finally, we are currently assessing a potential proliferative role of p38 in FGFR3-mutated bladder carcinomas (unpublished preliminary data), which might lead to further model refinement.

To wrap up, the present study demonstrates how Boolean modelling can recapitulate salient dynamical properties of an extremely complex biological network. As further details are uncovered, our logical model could be refined and eventually translated into a more quantitative framework (e.g. using ODEs or stochastic equations). In a first step towards more quantitative simulations, a continuous time Boolean framework could be used to explicitly represent time dependencies [81]. Tentatively, this approach would allow us to recapitulate more precisely the differential effects of transient versus sustained ERK activation [33], [37], [82].

Combining the delineation of a detailed reaction map and that of a predictive logical model, this study can serve as a basis to develop (semi-)automatic tools to derive logical models from reaction maps. Indeed, the manual derivation of a logical model from a complex reaction map presents risks of misinterpretations of either map symbols or map annotations. Errors are particularly likely to happen when the model is not built by the author of the map. In this respect, recent rule-based languages used to derive more quantitative models could be used to systematically derive predictive logical models, although potentially at the cost of additional efforts to build reaction maps in a more rigorous fashion [83]–[85].

## Supporting Information

Dataset S1 MAPK reaction map. The png (map) and txt (annotations) files were directly exported from the corresponding CellDesigner file (Dataset S2). Map components are coloured to emphasise relevant classes of proteins. The default protein colour is light green, whereas the default gene colour is yellow. MAPK cascades are coloured with different blue gradations (from light to dark blue going down the cascade). Scaffold proteins are coloured in darker green; phosphatases are coloured in red. Complete graphical notations can be found at www.celldesigner.org.
(ZIP)

Dataset S2 CellDesigner file (xml format) of the MAPK reaction map. Species and reactions are annotated and identifiers of the corresponding sources of information (PubMed IDs) are provided.
(XML)

Dataset S3 Summary of the results of the main simulations performed in this work. The xls file includes three sheets, erresponding to a model reduction. For each simulation, we report here the simulation ID (referenced in the main text and in the model files provided in Dataset S4), the perturbations performed (e.g. “EGFRgain; p53loss” indicates that EGFR was set to 1 and p53 was set to 0 throughout the simulation), the inputs considered and the initials states (asterisks denote all possible combinations of initial states). The attractor types (along with the number of corresponding states within parentheses, in the case of cyclic attractors) are further reported, as well as the corresponding component values in the attractor: 1 or 0 for stable values; asterisks for oscillating values.
(XLS)

Dataset S4 GINsim model files. For each model version (the original large model, and the three reduced versions), a file is provided in the format (zginml) that can be opened with the software GINsim (http://ginsim.org/beta). Simulation parameters have been encoded to ease the reproduction of the experiments referenced in the main text.
(ZIP)

Table S1 Selected MAPK modelling studies.
(PDF)

Table S2 Logical rules for the MAPK comprehensive model. & = AND; | = OR; ! = NOT. More details about modelling assumptions and references are provided in Table S4 (model documentation).
(PDF)

Table S3 Reduced MAPK models. We considered three alternative reductions of the MAPK model (columns), each preserving the input and phenotype components. Additional components (Selected observables) were kept depending on the simulations performed. The last row lists components that were conserved because they turned out to be auto-regulated at some point during the reduction procedure. Such auto-regulations arise from the compression of longer circuits.
(PDF)

Table S4 MAPK model documentation. Following a brief general description, all the components of the MAPK model (comprehensive version) are reported, along with their corresponding logical rules and annotations, including modelling hypotheses and links to the main sources of information (PubMed and HGNC databases).
(PDF)

Text S1 Supplementary text encompassing two sections. The first one describes how we derived the logical model from the reaction map. The second one demonstrates how we checked that all cyclic attractors obtained for the MAPK model reductions indeed correspond to attractors of the original comprehensive model.
(PDF)

Text S2 Hierarchical transition graphs associated with receptor alterations. Model dynamics following either EGFR over-expression or FGFR3 activating mutation (with all inputs set to 0, throughout the simulations) are depicted in two separated graphs, which were obtained using the reduced model version red1. For sake of simplicity, simulations were performed by using a single initial state with all the remaining variables set to 0 (the salient dynamics were preserved in these cases – cf. Dataset S3). The resulting hierarchical transition graphs (see Methods) are composed by different classes of nodes, emphasising strongly connected components (blue), and linear (non circular) pathways (pink). The attractors reached are represented at the bottom of the figures. Attractor colours refer to the corresponding phenotypes: red for proliferation, green for apoptosis, grey for no decision. Stables states are denoted by rectangles, while cyclic attractors are denoted by circles. The accompanying tables give the composition of each node of the corresponding HTG. For instance, the node cc1 of the HTG obtained for FGFR3 activating mutation corresponds to a strongly connected component of the state transition graph. The number of states belonging to it (i.e. 24), as well as the list of these states are listed in the table (asterisks denote all possible values for the corresponding variable).
(PDF)
