# Correction: Integrative Modelling of the Influence of MAPK Network on Cancer Cell Fate Decision

**DOI:** 10.1371/annotation/90e5e4be-952b-42b8-b56d-46baae3479ed

**Published:** 2013-11-18

**Authors:** Luca Grieco, Laurence Calzone, Isabelle Bernard-Pierrot, François Radvanyi, Brigitte Kahn-Perlès, Denis Thieffry

An error led to part of Figure 2 being removed, breaking the red line that should have linked the grey circles “PKC” and “EGFR”. The correct version of Figure 2 is now displayed on both the PDF and HTML versions of the article.

