# Supplementary figures and images for: Integrative Modelling of the Influence of MAPK Network on Cancer Cell Fate Decision
Source: PLoS Comput Biol. 2013 Oct 24;9(10):e1003286. doi: 10.1371/journal.pcbi.1003286 (PMC3821540; doi:10.1371/journal.pcbi.1003286)

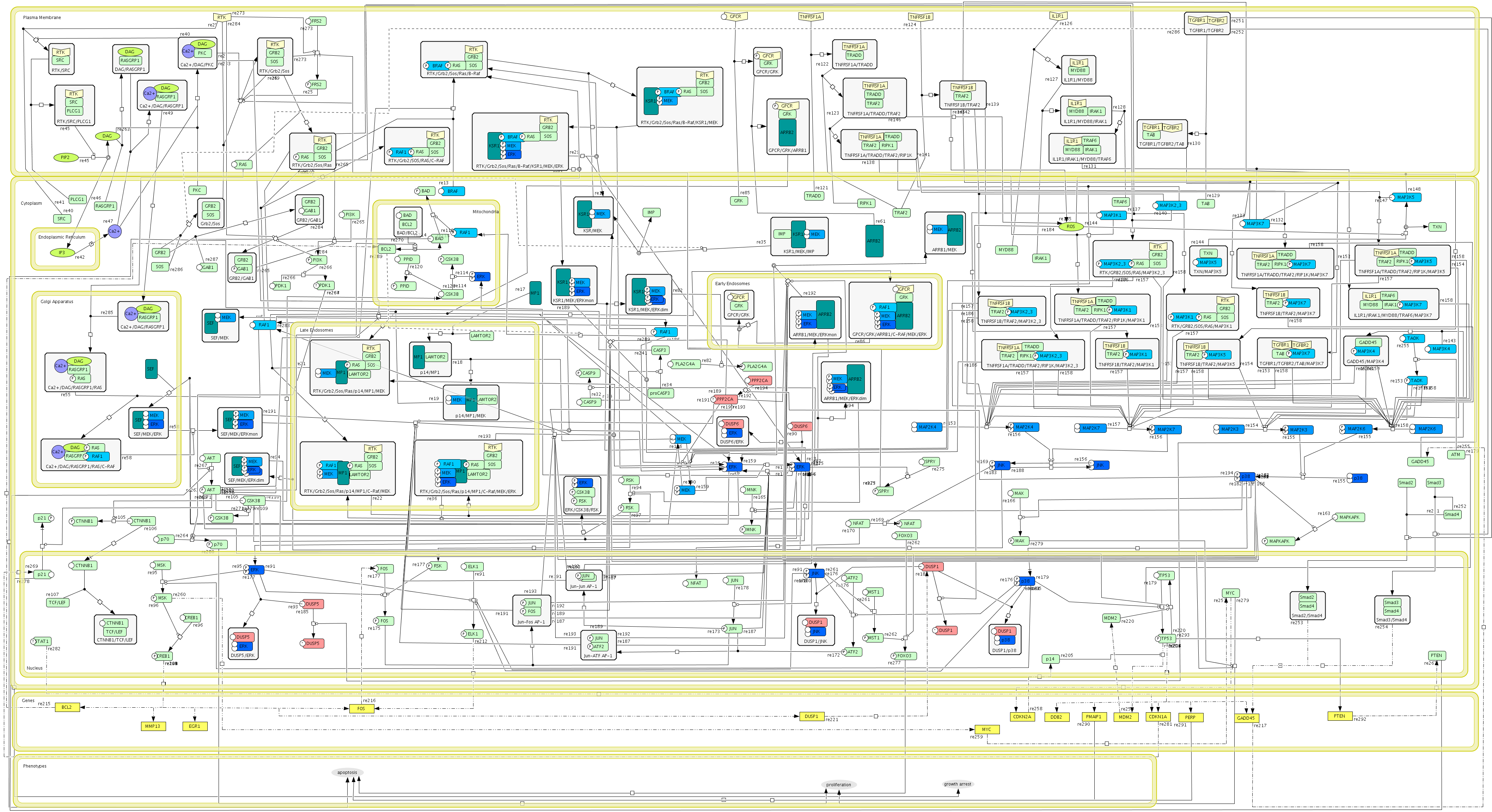

Supplement: Dataset S1 — MAPK reaction map. The png (map) and txt (annotations) files were directly exported from the corresponding CellDesigner file (Dataset S2). Map components are coloured to emphasise relevant classes of proteins. The default protein colour is light green, whereas the default gene colour is yellow. MAPK cascades are coloured with different blue gradations (from light to dark blue going down the cascade). Scaffold proteins are coloured in darker green; phosphatases are coloured in red. Complete graphical notations can be found at www.celldesigner.org. (ZIP) [file pcbi.1003286.s001.zip › MAPK_reaction_map_19june2013.png]
