# Supplementary material for: Integrative Modelling of the Influence of MAPK Network on Cancer Cell Fate Decision
Source: PLoS Comput Biol. 2013 Oct 24;9(10):e1003286. doi: 10.1371/journal.pcbi.1003286 (PMC3821540; doi:10.1371/journal.pcbi.1003286)
Supplement: Table S1 — Selected MAPK modelling studies. (PDF) [file pcbi.1003286.s005.pdf]

Authors	Formalism	Dimension	Description
Huang and Ferrel [26]	ODE	18	Ultrasensitivity of MAPK cascades depending on their particular structure: double phosphorylation and double de-phosphorylation mechanisms.
Kholodenko [27]	ODE	8	Sustained oscillations of MAPK phosphorylation level, induced by negative feedback loop and cascade ultrasensitivity.
Levchenko et al. [28]	ODE	27	Effects of scaffold proteins on MAPK activation.
Brightman and Fell [33]	ODE	29	Transient versus sustained ERK activation following growth signals.
Schoeberl et al. [34]	ODE	94	Dynamics of EGF-dependent ERK activation.
Hatakeyama et al. [35]	ODE	33	Interplay between PI3K-AKT and RAF-ERK pathways in EGFR signalling network.
Markevich et al. [29]	ODE	13, 15	Bistable behaviour of MAPK cascades due to multistep phosphorylation/de-phosphorylation cycles.
Hornberg et al. [36]	ODE	103	Identification of reactions controlling amplitude, duration and integrated output of EGF-dependent ERK signalling network.
Samaga et al. [43]	Logical model	104	Topological properties and qualitative behaviour of the EGFR signalling network.
Orton et al. [38]	ODE	31	Effects of cancerous alterations on EGFR/ERK pathway.
Sasagawa et al. [37]	ODE	22	Transient versus sustained ERK activation following growth signals.
Legewie et al. [30]	ODE	12, 21	Bistability of ERK activation due to MEK-ERK positive feedback circuit.
Smolen et al. [31]	ODE	26	Mechanisms contributing to the bistable behaviour of MAPK cascades.
Chen et al. [39]	ODE	478	Model of immediate-early signalling involving ErbB receptors, along with MAPK and PI3K pathways, trained against time course experimental data.
Sturm et al. [32]	ODE	14, 9	Negative feedback amplifier properties of ERK cascade.
Bagheri et al. [40]	ODE	4	Predictions of MEK inhibition-based treatment effects on tumour cell proliferation.
Finch et al. [42]	ODE	6	Phosphatase-mediated cross-talk between p38 and ERK cascades.
Handorf and Klipp [41]	Logical model	107	Boolean model of cross-talk between WNT and ERK pathways, semi-automatically derived from a public reaction database.
Poltz and Naumann [45]	Logical model	96	DNA damage response network in human epithelial tumours; identification of target molecules for DNA damaging therapies.

Table S1. Selected MAPK modelling studies. ODE = ordinary differential equations.
