# Supplementary material for: Integrative Modelling of the Influence of MAPK Network on Cancer Cell Fate Decision
Source: PLoS Comput Biol. 2013 Oct 24;9(10):e1003286. doi: 10.1371/journal.pcbi.1003286 (PMC3821540; doi:10.1371/journal.pcbi.1003286)
Supplement: Table S3 — Reduced MAPK models. We considered three alternative reductions of the MAPK model (columns), each preserving the input and phenotype components. Additional components (Selected observables) were kept depending on the simulations performed. The last row lists components that were conserved because they turned out to be auto-regulated at some point during the reduction procedure. Such auto-regulations arise from the compression of longer circuits. (PDF) [file pcbi.1003286.s007.pdf]

	Model versions		
	**red1**	**red2**	**red3**
**Inputs**	EGFR_stimulus, FGFR3_stimulus, TGFBR_stimulus, DNA_damage		
**Phenotypes**	Proliferation, Apoptosis, Growth_Arrest		
**Selected observables**	EGFR, FGFR3, p53, p14, PI3K, AKT, PTEN, ERK	EGFR, FGFR3, RAF, RAS, ERK, AKT, p53, p21	JNK, p38, GADD45, ERK, RAS
**Additional auto-regulated components**	FRS2, MSK	GRB2, PI3K, p38	GRB2, PLCG, PI3K, MDM2

Table S3. Reduced MAPK models. We considered three alternative reductions of the MAPK model (columns), each preserving the input and phenotype components. Additional components (Selected observables) were kept depending on the simulations performed. The last row lists components that were conserved because they turned out to be auto-regulated at some point during the reduction procedure. Such auto-regulations arise from the compression of longer circuits.
