# Supplementary material for: Integrative Modelling of the Influence of MAPK Network on Cancer Cell Fate Decision
Source: PLoS Comput Biol. 2013 Oct 24;9(10):e1003286. doi: 10.1371/journal.pcbi.1003286 (PMC3821540; doi:10.1371/journal.pcbi.1003286)
Supplement: Table S4 — MAPK model documentation. Following a brief general description, all the components of the MAPK model (comprehensive version) are reported, along with their corresponding logical rules and annotations, including modelling hypotheses and links to the main sources of information (PubMed and HGNC databases). (PDF) [file pcbi.1003286.s008.pdf]

Annotations of the model "MAPK_large_19june2013"

<table>
<tr><th>Nodes</th><th>Logical rules</th><th>Annotations</th></tr>
<tr><td>Apoptosis</td><td>!BCL2 & !ERK & FOXO3 & p53</td><td>http://www.ncbi.nlm.nih.gov/pubmed/20978166
http://www.ncbi.nlm.nih.gov/pubmed/19641508
http://www.ncbi.nlm.nih.gov/pubmed/12792650
http://www.ncbi.nlm.nih.gov/pubmed/12040186
Phenotype

VARIABLE: apoptosis enablement
Modelling hypotheses: apoptosis is supposed to be enabled when at least a pro-apoptotic component is activated (p53, FOXO3) and no anti-apoptotic components are activated (ERK, BCL2).
FOXO3a promotes apoptosis through activation of PUMA.
PMID:20978166
In response to genotoxic stress such as DNA damage, PUMA is transactivated by p53 (leading to apoptosis).
PMID:19641508
Caspase 9 is efficiently phosphorylated on Thr125 by ERK in vitro, suggesting that it is targeted directly by ERK in vivo. This is one of the ways ERK plays its anti-apoptotic role.
PMID:12792650
Akt phosphorylation of the apoptosis-inducing protein Bad creates a binding site for 14-3-3 proteins and prevents Bad from binding to Bcl-2 family members Bcl-2 and Bcl-XL, thus releasing them for a cell survival response.
PMID:12040186

<table>
<tr><th>Regulator</th><th>Comment</th></tr>
<tr><td>ERK</td><td>http://www.ncbi.nlm.nih.gov/pubmed/12792650
Caspase 9 is efficiently phosphorylated on Thr125 by ERK in vitro, suggesting that it is targeted directly by ERK in vivo. This is one of the ways ERK plays its anti-apoptotic role.
PMID:12792650</td></tr>
<tr><td>p53</td><td>http://www.ncbi.nlm.nih.gov/pubmed/19641508
In response to genotoxic stress such as DNA damage, PUMA is transactivated by p53 (leading to apoptosis).
PMID:19641508</td></tr>
<tr><td>FOXO3</td><td>http://www.ncbi.nlm.nih.gov/pubmed/20978166
FOXO3a promotes apoptosis through activation of PUMA.
PMID:20978166</td></tr>
<tr><td>BCL2</td><td>http://www.ncbi.nlm.nih.gov/pubmed/12040186</td></tr>
</table>
</td></tr>
</table>

			Akt phosphorylation of the apoptosis-inducing protein Bad creates a binding site for 14-3-3 proteins and prevents Bad from binding to Bcl-2 family members Bcl-2 and Bcl-XL, thus releasing them for a cell survival response.<br>PMID:12040186
AKT	PDK1 & !PTEN	http://www.genenames.org/data/hgnc_data.php?hgnc_id=391<br>http://www.genenames.org/data/hgnc_data.php?hgnc_id=392<br>http://www.genenames.org/data/hgnc_data.php?hgnc_id=393<br>http://www.ncbi.nlm.nih.gov/pubmed/15567848<br>http://www.ncbi.nlm.nih.gov/pubmed/12040186<br>http://www.ncbi.nlm.nih.gov/pubmed/11882383<br>http://www.ncbi.nlm.nih.gov/pubmed/10869359<br>http://www.ncbi.nlm.nih.gov/pubmed/11850850<br>http://www.ncbi.nlm.nih.gov/pubmed/12217521<br>AKT1 = v-akt murine thymoma viral oncogene homolog 1<br>AKT2 = v-akt murine thymoma viral oncogene homolog 2<br>AKT3 = v-akt murine thymoma viral oncogene homolog 3<br>VARIABLE: AKT (any isoform) phosphorylation level<br>Grb2 recruits the docking protein Gab1, which is tyrosine phosphorylated by FGFR, leading to recruitment and activation of the PI3K-Akt cell survival pathway.<br>EGF stimulates a cell survival pathway mediated by phosphoinositide 3-kinase (PI3K) and the protein kinase Akt by an indirect mechanism in which tyrosine phosphorylation of the docking protein Gab1 or ErbB3 by EGFR leads to recruitment and activation of PI3K.<br>PMID:15567848<br>Signaling proteins with pleckstrin-homology (PH) domains accumulate at sites of PI3K activation by directly binding to PI(3,4,5)P3. Of particular interest are the protein serine-threonine kinases Akt and phosphoinositide-dependent kinase 1 (PDK1). Association with PI(3,4,5)P3 at the membrane brings these proteins into proximity and facilitates phosphorylation of Akt by PDK1. This phosphorylation stimulates the catalytic activity of Akt, resulting in the phosphorylation of a host of other proteins that affect cell growth, cell cycle entry, and cell survival.<br>Akt phosphorylation of the apoptosis-inducing protein Bad creates a binding site for 14-3-3 proteins and prevents Bad from binding to Bcl-2 family members Bcl-2 and Bcl-XL, thus releasing them for a cell survival response.<br>Glycogen synthase kinase 3 (GSK3) is a target of Akt. This protein kinase is constitutively active in unstimulated cells and phosphorylates many proteins (including glycogen synthase, c-Myc, and cyclin D) to keep them in inactive states or promote their degradation. Phosphorylation of GSK3 (both alpha and beta isoforms) by Akt turns off the catalytic activity of this enzyme, resulting in the activation of pathways that are normally repressed by GSK3.<br>Phosphorylation of FOXO3 by Akt creates a binding site for the 14-3-3 family of proteins. The complex of FOXO3 and 14-3-3 is retained in the cytosol, blocking transcription of genes normally stimulated by FOXO3.<br>PMID:12040186<br>p21 is a direct substrate of AKT and that this may regulate the subcellular localisation of p21. Phosphorylation of p21	

<table>
<tr><td></td><td></td><td>by AKT correlated with its exit from the nucleus and with increased cell cycle progression.
PMID:11882383
Phosphorylation by Akt inhibits EGF-dependent activity of B-Raf and C-Raf.
PMID:10869359
Effective recruitment of Akt by appropriate survival signals may lead to activation of Mdm2, inactivation of p53, and eventually inhibition of p53-dependent apoptosis.
PMID:11850850
p53 induces transcription of the PTEN gene and elevates cellular levels of the PTEN protein, which in turn inhibits activation of Akt.
PMID:12217521

<table>
<tr><th>Regulator</th><th>Comment</th></tr>
<tr><td>PTEN</td><td>http://www.ncbi.nlm.nih.gov/pubmed/12217521
p53 induces transcription of the PTEN gene and elevates cellular levels of the PTEN protein, which in turn inhibits activation of Akt.
PMID:12217521</td></tr>
<tr><td>PDK1</td><td>http://www.ncbi.nlm.nih.gov/pubmed/12040186
Signaling proteins with pleckstrin-homology (PH) domains accumulate at sites of PI3K activation by directly binding to PI(3,4,5)P3. Of particular interest are the protein serine-threonine kinases Akt and phosphoinositide-dependent kinase 1 (PDK1). Association with PI(3,4,5)P3 at the membrane brings these proteins into proximity and facilitates phosphorylation of Akt by PDK1. This phosphorylation stimulates the catalytic activity of Akt, resulting in the phosphorylation of a host of other proteins that affect cell growth, cell cycle entry, and cell survival.
PMID:12040186</td></tr>
</table></td></tr>
<tr><td>AP1</td><td>JUN & (FOS | ATF2)</td><td>http://www.ncbi.nlm.nih.gov/pubmed/11274345
http://www.ncbi.nlm.nih.gov/pubmed/18039929
AP-1
VARIABLE: AP-1 formation
AP-1 is a heterodimer comprised of bZIP transcription factors, typically c-Jun and JunD, along with members of the fos (usually c-Fos) and ATF (usually ATF2) families. All bZIP transcription factors contain leucine zippers that enable homo- and heterodimerisation, and AP-1 components are organised into Jun-Jun, Jun-Fos, or Jun-ATF dimers.
PMID:11274345
AP-1 mediated gene expression inhibits ERK phosphorylation.
PMID:18039929</td></tr>
</table>

<table>
<tr>
<td></td>
<td></td>
<td>
<table>
<tr><th>Regulator</th><th>Comment</th></tr>
<tr><td>JUN</td><td>http://www.ncbi.nlm.nih.gov/pubmed/11274345
AP-1 is a heterodimer comprised of bZIP transcription factors, typically c-Jun and JunD, along with members of the fos (usually c-Fos) and ATF (usually ATF2) families. All bZIP transcription factors contain leucine zippers that enable homo- and heterodimerisation, and AP-1 components are organised into Jun-Jun, Jun-Fos, or Jun-ATF dimers.
PMID:11274345</td></tr>
<tr><td>ATF2</td><td>http://www.ncbi.nlm.nih.gov/pubmed/11274345
AP-1 is a heterodimer comprised of bZIP transcription factors, typically c-Jun and JunD, along with members of the fos (usually c-Fos) and ATF (usually ATF2) families. All bZIP transcription factors contain leucine zippers that enable homo- and heterodimerisation, and AP-1 components are organised into Jun-Jun, Jun-Fos, or Jun-ATF dimers.
PMID:11274345</td></tr>
<tr><td>FOS</td><td>http://www.ncbi.nlm.nih.gov/pubmed/11274345
AP-1 is a heterodimer comprised of bZIP transcription factors, typically c-Jun and JunD, along with members of the fos (usually c-Fos) and ATF (usually ATF2) families. All bZIP transcription factors contain leucine zippers that enable homo- and heterodimerisation, and AP-1 components are organised into Jun-Jun, Jun-Fos, or Jun-ATF dimers.
PMID:11274345</td></tr>
</table>
</td>
</tr>
<tr>
<td>ATF2</td>
<td>JNK | p38</td>
<td>http://www.genenames.org/data/hgnc_data.php?hgnc_id=784
http://www.ncbi.nlm.nih.gov/pubmed/11274345
ATF2 = activating transcription factor 2
VARIABLE: ATF2 phosphorylation level
AP-1 is a heterodimer comprised of bZIP transcription factors, typically c-Jun and JunD, along with members of the fos (usually c-Fos) and ATF (usually ATF2) families. All bZIP transcription factors contain leucine zippers that enable homo- and heterodimerisation, and AP-1 components are organised into Jun-Jun, Jun-Fos, or Jun-ATF dimers.
JNK and p38 can both phosphorylate ATF2 at Thr69 and Ser71.
PMID:11274345
<table>
<tr><th>Regulator</th><th>Comment</th></tr>
</table>
</td>
</tr>
</table>

<table>
<tr><td></td><td></td><td><table><tr><td>JNK</td><td>http://www.ncbi.nlm.nih.gov/pubmed/11274345
JNK and p38 can both phosphorylate ATF2 at Thr69 and Ser71.
PMID:11274345</td></tr><tr><td>p38</td><td>http://www.ncbi.nlm.nih.gov/pubmed/11274345
JNK and p38 can both phosphorylate ATF2 at Thr69 and Ser71.
PMID:11274345</td></tr></table></td></tr>
<tr><td>ATM</td><td>DNA_damage</td><td>http://www.genenames.org/data/hgnc_data.php?hgnc_id=795
http://www.ncbi.nlm.nih.gov/pubmed/18235226
http://www.ncbi.nlm.nih.gov/pubmed/18855897
http://www.ncbi.nlm.nih.gov/pubmed/15140942
ATM = ataxia telangiectasia mutated
VARIABLE: ATM phosphorylation level
ATM autophosphorylates itself at serine 1981 in response to DNA damage and some other cellular stresses.
PMID:18235226
Ataxia telangiectasia mutated (ATM) is activated in response to DNA damage and directly phosphorylates TAOK.
PMID:18855897
ATM phosphorylates p53 at ser15 and stabilize it.
PMID:15140942
<table><tr><th>Regulator</th><th>Comment</th></tr><tr><td>DNA_damage</td><td>http://www.ncbi.nlm.nih.gov/pubmed/18235226
ATM autophosphorylates itself at serine 1981 in response to DNA damage and some other cellular stresses.
PMID:18235226</td></tr></table></td></tr>
<tr><td>BCL2</td><td>CREB & AKT</td><td>http://www.genenames.org/data/hgnc_data.php?hgnc_id=990
http://www.ncbi.nlm.nih.gov/pubmed/19815709
http://www.ncbi.nlm.nih.gov/pubmed/12040186
BCL2 = B-cell CLL/lymphoma 2
VARIABLE: BCL2 activation level (implies gene expression)
Modelling hypotheses: CREB is needed for BCL2 gene expression, AKT is needed for BCL2 activation.
DUSP1, FOS, BCL2 are CREB target genes.
PMID:19815709
Akt phosphorylation of the apoptosis-inducing protein Bad creates a binding site for 14-3-3 proteins and prevents Bad from binding to Bcl-2 family members Bcl-2 and Bcl-XL, thus releasing them for a cell survival response.
PMID:12040186
<table><tr><th>Regulator</th><th>Comment</th></tr><tr><td>CREB</td><td>http://www.ncbi.nlm.nih.gov/pubmed/19815709</td></tr></table></td></tr>
</table>

		AKT — DUSP1, FOS and BCL2 are CREB target genes.<br>PMID:19815709<br>http://www.ncbi.nlm.nih.gov/pubmed/12040186<br>Akt phosphorylation of the apoptosis-inducing protein Bad creates a binding site for 14-3-3 proteins and prevents Bad from binding to Bcl-2 family members Bcl-2 and Bcl-XL, thus releasing them for a cell survival response.<br>PMID:12040186
CREB	MSK	http://www.genenames.org/data/hgnc_data.php?hgnc_id=2345<br>http://www.ncbi.nlm.nih.gov/pubmed/15187187<br>http://www.ncbi.nlm.nih.gov/pubmed/19815709<br>CREB1 = cAMP responsive element binding protein 1<br>VARIABLE: CREB phosphorylation level<br>MSK1 mediates the mitogen-stimulated phoshphorylation of CREB.<br>PMID:15187187<br>DUSP1, FOS and BCL2 are CREB target genes.<br>PMID:19815709<br><br>**Regulator** — **Comment**<br>MSK — http://www.ncbi.nlm.nih.gov/pubmed/15187187<br>MSK1 mediates the mitogen-stimulated phoshphorylation of CREB.<br>PMID:15187187
DNA_damage	input	http://www.ncbi.nlm.nih.gov/pubmed/18235226<br>Model input<br>VARIABLE: DNA damaging agents/therapies action<br>ATM autophosphorylates itself at serine 1981 in response to DNA damage and some other cellular stresses.<br>PMID:18235226
DUSP1	CREB	http://www.genenames.org/data/hgnc_data.php?hgnc_id=3064<br>http://www.ncbi.nlm.nih.gov/pubmed/19815709<br>http://www.ncbi.nlm.nih.gov/pubmed/19436832<br>DUSP1 = dual specificity phosphatase 1<br>VARIABLE: DUSP1 gene expression level<br>DUSP1 is a CREB target gene.<br>PMID:19815709<br>DUSP1 preferentially inactivates JNK and p38.<br>PMID:19436832

<table>
<tr><td></td><td></td><td><table><tr><th>Regulator</th><th>Comment</th></tr><tr><td>CREB</td><td>http://www.ncbi.nlm.nih.gov/pubmed/19815709
DUSP1, FOS and BCL2 are CREB target genes.
PMID:19815709</td></tr></table></td></tr>
<tr><td>EGFR</td><td>(EGFR_stimulus | SPRY) & !(PKC | GRB2)</td><td>http://www.genenames.org/data/hgnc_data.php?hgnc_id=3236
http://www.ncbi.nlm.nih.gov/pubmed/15567848
http://www.ncbi.nlm.nih.gov/pubmed/15173823
http://www.ncbi.nlm.nih.gov/pubmed/6321473
http://www.ncbi.nlm.nih.gov/pubmed/17496910
http://www.ncbi.nlm.nih.gov/pubmed/2472219
EGFR = epidermal growth factor receptor
VARIABLE: EGFR activation level
The ubiquitin ligase Cbl interacts with EGFR directly and indirectly through Grb2, promoting ubiquitination and degradation of EGFR
PMID:15567848
Spry is induced by activated ERK. It positively regulates EGFR signalling by sequestering Cbl (positive feedback in the model, from Spry to EGFR), whereas it negatively regulates FGFR signalling by sequestering Grb2 from FSR2 (negative feedback in the model, from Spry to FSR2).
PMID:15173823
PKC may cause phosphorylation of EGFR, leading to a decrease in its activity.
PMID:6321473
Grb2 is an adaptor protein, normally present in cytosol. It is recruited to the plasma membrane by activated RTKs.
PMID:17496910
Tyrosine phosphorylation of PLC-gamma by EGF receptor leads to its activation.
PMID:2472219
<table><tr><th>Regulator</th><th>Comment</th></tr><tr><td>GRB2</td><td>http://www.ncbi.nlm.nih.gov/pubmed/15567848
The ubiquitin ligase Cbl interacts with EGFR directly and indirectly through Grb2, promoting ubiquitination and degradation of EGFR
PMID:15567848</td></tr><tr><td>SPRY</td><td>http://www.ncbi.nlm.nih.gov/pubmed/15173823
Spry is induced by activated ERK. It positively regulates EGFR signalling by sequestering Cbl (positive feedback in the model, from Spry to EGFR), whereas it negatively regulates FGFR signalling by sequestering Grb2 from FSR2 (negative feedback in the model, from Spry to FSR2).
PMID:15173823</td></tr></table></td></tr>
</table>

		PKC	http://www.ncbi.nlm.nih.gov/pubmed/6321473<br>PKC may cause phosphorylation of EGFR, leading to a decrease in its activity.<br>PMID:6321473
EGFR_stimulus	input	Model input<br>VARIABLE: any stimulus able to induce EGFR activation	
ELK1	ERK | JNK | p38	http://www.genenames.org/data/hgnc_data.php?hgnc_id=3321<br>http://www.ncbi.nlm.nih.gov/pubmed/16393692<br>http://www.ncbi.nlm.nih.gov/pubmed/20506250<br>http://www.ncbi.nlm.nih.gov/pubmed/11274345<br>ELK1 = ELK1, member of ETS oncogene family<br>VARIABLE: ELK1 phosphorylation level<br>The rapid and efficient phosphorylation of Elk1 by ERKs is enabled by a direct interaction between the two proteins.<br>PMID:16393692<br>ELK1 is a nuclear p38 target.<br>PMID:20506250<br>ELK1 is a nuclear JNK target.<br>ELK1 induces c-fos expression.<br>PMID:11274345<br>**Regulator**<br>JNK<br><br><br>p38	 <br><br><br><br><br><br><br><br><br><br><br><br>**Comment**<br>http://www.ncbi.nlm.nih.gov/pubmed/11274345<br>ELK1 is a nuclear JNK target.<br>PMID:11274345<br>http://www.ncbi.nlm.nih.gov/pubmed/20506250<br>ELK1 is a nuclear p38 target.<br>PMID:20506250
ERK	MEK1_2	http://www.genenames.org/data/hgnc_data.php?hgnc_id=6871<br>http://www.genenames.org/data/hgnc_data.php?hgnc_id=6877<br>http://www.ncbi.nlm.nih.gov/pubmed/17496910<br>http://www.ncbi.nlm.nih.gov/pubmed/18039929<br>http://www.ncbi.nlm.nih.gov/pubmed/15173823<br>http://www.ncbi.nlm.nih.gov/pubmed/12792650<br>http://www.ncbi.nlm.nih.gov/pubmed/15187187<br>http://www.ncbi.nlm.nih.gov/pubmed/15239952<br>http://www.ncbi.nlm.nih.gov/pubmed/16393692<br>http://www.ncbi.nlm.nih.gov/pubmed/11940578<br>http://www.ncbi.nlm.nih.gov/pubmed/10601235<br>http://www.ncbi.nlm.nih.gov/pubmed/11431469<br>MAPK1 = mitogen-activated protein kinase 1	

<table>
<tr><td></td><td></td><td>MAPK3 = mitogen-activated protein kinase 3
VARIABLE: ERK (any isoform) phosphorylation level
Once activated, Raf family members are capable of initiating the phosphorylation cascade, whereby Raf activates MEK, and MEK in turn activates ERK.
PMID:17496910
Spry is induced by activated ERK, through phosphorylation on Tyr55.
PMID:15173823
Caspase 9 is efficiently phosphorylated on Thr125 by ERK in vitro, suggesting that it is targeted directly by ERK in vivo. This is one of the ways ERK plays its anti-apoptotic role.
PMID:12792650
MSK1 and MSK2 are potently activated (by phosphorylation) in vivo by ERK1/2 and p38 but not JNK.
RSKs interact with ERK and dissociate upon activation.
PMID:15187187
RSK2 is a well known ERK substrate in the cytoplasm and has been shown to undergo autophosphorylation after ERK phosphorylation.
PMID:15239952
Phosphorylation by both ERKs and their downstream RSKs can stabilise the c-Fos protein for several hours. The combination of these phosphorylations allows c-Fos sustained activity.
The rapid and efficient phosphorylation of Elk1 by ERKs is enabled by a direct interaction between the two proteins.
PMID:16393692
p70 activation requires both ERK cascade and PI3K/AKT cascade, at least in some cell types.
PMID:11940578, PMID:10601235, PMID:11431469
<table><tr><th>Regulator</th><th>Comment</th></tr><tr><td>MEK1_2</td><td>http://www.ncbi.nlm.nih.gov/pubmed/17496910
Once activated, Raf family members are capable of initiating the phosphorylation cascade, whereby Raf activates MEK, and MEK in turn activates ERK.
PMID:17496910</td></tr></table></td></tr>
<tr><td>FGFR3</td><td>FGFR3_stimulus & !(GRB2 | PKC)</td><td>http://www.genenames.org/data/hgnc_data.php?hgnc_id=3690
http://www.ncbi.nlm.nih.gov/pubmed/15567848
http://www.ncbi.nlm.nih.gov/pubmed/11447289
http://www.ncbi.nlm.nih.gov/pubmed/1656221
http://www.ncbi.nlm.nih.gov/pubmed/9694798
http://www.ncbi.nlm.nih.gov/pubmed/8622701
http://www.ncbi.nlm.nih.gov/pubmed/14699054
FGFR3 = fibroblast growth factor receptor 3
VARIABLE: FGFR3 activation level
Grb2, bound to Cbl, does not interact directly with FGFR but, rather, binds to tyrosine-phosphorylated FRS2, promoting ubiquitination and degradation of FRS2 and FGFR</td></tr>
</table>

<table>
<tr><td></td><td></td><td>PMID:15567848
FGF stimulation leads to phosphorylation of Shp2 on a tyrosine residue that forms a complex with an additional molecule of Grb2. Grb2/Sos complexes are thus recruited directly and indirectly via Shp2 upon tyrosine phosphorylation of FRS2a in response to growth factor stimulation.
PMID:11447289
Binding of FGF to FGFR leads to tyrosine phosphorylation of PLCgamma.
PMID:1656221
(Inferred from FGFR1) PKC-mediated phosphorylation can lead to internalization and degradation of FGFR1.
PMID:9694798, PMID:8622701, PMID:14699054

<table>
<tr><th>Regulator</th><th>Comment</th></tr>
<tr><td>GRB2</td><td>http://www.ncbi.nlm.nih.gov/pubmed/11997436
FGF-induced tyrosine phosphorylation of FRS2 results in complex formation with the adaptor protein Grb2 bound to Cbl by means of its SH3 domains. FGF-induced ternary complex formation among FRS2, Grb2, and Cbl results in ubiquitination and degradation of FRS2 and FGF receptor (FGFR).
PMID:11997436</td></tr>
<tr><td>PKC</td><td>http://www.ncbi.nlm.nih.gov/pubmed/9694798
http://www.ncbi.nlm.nih.gov/pubmed/8622701
http://www.ncbi.nlm.nih.gov/pubmed/14699054
(Inferred from FGFR1) PKC-mediated phosphorylation can lead to internalization and degradation of FGFR1.
PMID:9694798, PMID:8622701, PMID:14699054</td></tr>
</table>
</td></tr>
<tr><td>FGFR3_stimulus</td><td>input</td><td>Model input
VARIABLE: any stimulus able to induce FGFR3 activation</td></tr>
<tr><td>FOS</td><td>ERK & RSK & (ELK1 | CREB)</td><td>http://www.genenames.org/data/hgnc_data.php?hgnc_id=3796
http://www.ncbi.nlm.nih.gov/pubmed/11274345
http://www.ncbi.nlm.nih.gov/pubmed/19815709
http://www.ncbi.nlm.nih.gov/pubmed/16393692
FOS = FBJ murine osteosarcoma viral oncogene homolog
VARIABLE: FOS phosphorylation level
Further observations: ELK1 or CREB are supposed to induce FOS gene expression, ERK or RSK are supposed to activate it
AP-1 is a heterodimer comprised of bZIP transcription factors, typically c-Jun and JunD, along with members of the fos (usually c-Fos) and ATF (usually ATF2) families. All bZIP transcription factors contain leucine zippers that enable homo- and heterodimerisation, and AP-1 components are organised into Jun-Jun, Jun-Fos, or Jun-ATF dimers.
ELK1 induces c-fos expression.
PMID:11274345</td></tr>
</table>

<table>
<tr><td></td><td></td><td>FOS is a CREB target gene.
PMID:19815709
Phosphorylation by both ERKs and their downstream RSKs can stabilise the c-Fos protein for several hours. The combination of these phosphorylations allows c-Fos sustained activity.
PMID:16393692

<table>
<tr><th>Regulator</th><th>Comment</th></tr>
<tr><td>ERK</td><td>http://www.ncbi.nlm.nih.gov/pubmed/16393692
Phosphorylation by both ERKs and their downstream RSKs can stabilise the c-Fos protein for several hours. The combination of these phosphorylations allows c-Fos sustained activity.
PMID:16393692</td></tr>
<tr><td>RSK</td><td>http://www.ncbi.nlm.nih.gov/pubmed/16393692
Phosphorylation by both ERKs and their downstream RSKs can stabilise the c-Fos protein for several hours. The combination of these phosphorylations allows c-Fos sustained activity.
PMID:16393692</td></tr>
<tr><td>ELK1</td><td>http://www.ncbi.nlm.nih.gov/pubmed/11274345
ELK1 induces c-fos expression.
PMID:11274345</td></tr>
<tr><td>CREB</td><td>http://www.ncbi.nlm.nih.gov/pubmed/19815709
DUSP1, FOS and BCL2 are CREB target genes.
PMID:19815709</td></tr>
</table></td></tr>
<tr><td>FOXO3</td><td>JNK & !AKT</td><td>http://www.genenames.org/data/hgnc_data.php?hgnc_id=3821
http://www.ncbi.nlm.nih.gov/pubmed/20028971
http://www.ncbi.nlm.nih.gov/pubmed/20978166
http://www.ncbi.nlm.nih.gov/pubmed/12040186
FOXO3 = forkhead box O3
VARIABLE: FOXO3 phosphorylation level
JNK phosphorylates MST1 at serine 82, which leads to the enhancement of MST1 activation. The activation of MST1 phosphorylates FOXO3 at serine 207 and promotes cell death.
PMID:20028971
FOXO3a promotes apoptosis through activation of PUMA.
PMID:20978166
Phosphorylation of FOXO3 by Akt creates a binding site for the 14-3-3 family of proteins. The complex of FOXO3 and 14-3-3 is retained in the cytosol, blocking transcription of genes normally stimulated by FOXO3.
PMID:12040186</td></tr>
</table>

<table>
<tr>
<td></td>
<td></td>
<td>
<table>
<tr><th>Regulator</th><th>Comment</th></tr>
<tr><td>JNK</td><td>http://www.ncbi.nlm.nih.gov/pubmed/20028971
JNK phosphorylates MST1 at serine 82, which leads to the enhancement of MST1 activation. The activation of MST1 phosphorylates FOXO3 at serine 207 and promotes cell death.
PMID:20028971</td></tr>
<tr><td>AKT</td><td>http://www.ncbi.nlm.nih.gov/pubmed/12040186
Phosphorylation of FOXO3 by Akt creates a binding site for the 14-3-3 family of proteins. The complex of FOXO3 and 14-3-3 is retained in the cytosol, blocking transcription of genes normally stimulated by FOXO3.
PMID:12040186</td></tr>
</table>
</td>
</tr>
<tr>
<td>FRS2</td>
<td>FGFR3 & !SPRY & !GRB2</td>
<td>http://www.genenames.org/data/hgnc_data.php?hgnc_id=16971
http://www.ncbi.nlm.nih.gov/pubmed/11447289
http://www.ncbi.nlm.nih.gov/pubmed/15173823
http://www.ncbi.nlm.nih.gov/pubmed/11997436
FRS2 = fibroblast growth factor receptor substrate 2
VARIABLE: FRS2 phosphorylation level
FGF stimulation leads to phosphorylation of Shp2 on a tyrosine residue that forms a complex with an additional molecule of Grb2. Grb2/Sos complexes are thus recruited directly and indirectly via Shp2 upon tyrosine phosphorylation of FRS2a in response to growth factor stimulation.
PMID:11447289
Spry is induced by activated ERK. It positively regulates EGFR signalling by sequestering Cbl (positive feedback in the model, from Spry to EGFR), whereas it negatively regulates FGFR signalling by sequestering Grb2 from FSR2 (negative feedback in the model, from Spry to FSR2).
PMID:15173823
FGF-induced tyrosine phosphorylation of FRS2 results in complex formation with the adaptor protein Grb2 bound to Cbl by means of its SH3 domains. FGF-induced ternary complex formation among FRS2, Grb2, and Cbl results in ubiquitination and degradation of FRS2 and FGF receptor (FGFR).
PMID:11997436
<table>
<tr><th>Regulator</th><th>Comment</th></tr>
<tr><td>GRB2</td><td>http://www.ncbi.nlm.nih.gov/pubmed/11997436
FGF-induced tyrosine phosphorylation of FRS2 results in complex formation with the adaptor protein Grb2 bound to Cbl by means of its SH3 domains. FGF-induced ternary complex formation among FRS2, Grb2, and Cbl results in ubiquitination and degradation of FRS2 and FGF receptor (FGFR).</td></tr>
</table>
</td>
</tr>
</table>

<table>
<tr>
<td></td>
<td></td>
<td>
<table>
<tr><td></td><td>PMID:11997436</td></tr>
<tr><td>SPRY</td><td>http://www.ncbi.nlm.nih.gov/pubmed/15173823
Spry is induced by activated ERK. It positively regulates EGFR signalling by sequestering Cbl (positive feedback in the model, from Spry to EGFR), whereas it negatively regulates FGFR signalling by sequestering Grb2 from FSR2 (negative feedback in the model, from Spry to FSR2).
PMID:15173823</td></tr>
<tr><td>FGFR3</td><td>http://www.ncbi.nlm.nih.gov/pubmed/11447289
FGF stimulation leads to phosphorylation of Shp2 on a tyrosine residue that forms a complex with an additional molecule of Grb2. Grb2/Sos complexes are thus recruited directly and indirectly via Shp2 upon tyrosine phosphorylation of FRS2a in response to growth factor stimulation.
PMID:11447289</td></tr>
</table>
</td>
</tr>
<tr>
<td>GAB1</td>
<td>GRB2 | PI3K</td>
<td>http://www.genenames.org/data/hgnc_data.php?hgnc_id=4066
http://www.ncbi.nlm.nih.gov/pubmed/15567848
http://www.ncbi.nlm.nih.gov/pubmed/15199124
GAB1 = GRB2-associated binding protein 1
VARIABLE: GAB1 phosphorylation level
Grb2 recruits the docking protein Gab1, which is tyrosine phosphorylated by EGFR or FGFR, leading to recruitment and activation of the PI3K-Akt cell survival pathway.
PMID:15567848
Recruitment of PI 3-kinase by Gab1 results in a positive-feedback loop mediated by binding of the PH domain of Gab1 to the product of PI3-kinase activation, phosphatidylinositol-3,4,5-triphosphate.
PMID:15199124
<table>
<tr><td>Regulator</td><td>Comment</td></tr>
<tr><td>GRB2</td><td>http://www.ncbi.nlm.nih.gov/pubmed/15567848
Grb2 recruits the docking protein Gab1, which is tyrosine phosphorylated by FGFR, leading to recruitment and activation of the PI3K-Akt cell survival pathway.
EGF stimulates a cell survival pathway mediated by phosphoinositide 3-kinase (PI3K) and the protein kinase Akt by an indirect mechanism in which tyrosine phosphorylation of the docking protein Gab1 or ErbB3 by EGFR leads to recruitment and activation of PI3K.
PMID:15567848</td></tr>
</table>
</td>
</tr>
</table>

		PI3K	http://www.ncbi.nlm.nih.gov/pubmed/15199124<br>Recruitment of PI 3-kinase by Gab1 results in a positive-feedback loop mediated by binding of the PH domain of Gab1 to the product of PI3-kinase activation, phosphatidylinositol-3,4,5-triphosphate.<br>PMID:15199124
GADD45	SMAD | p53	http://www.genenames.org/data/hgnc_data.php?hgnc_id=4095<br>http://www.genenames.org/data/hgnc_data.php?hgnc_id=4096<br>http://www.genenames.org/data/hgnc_data.php?hgnc_id=4097<br>http://www.ncbi.nlm.nih.gov/pubmed/21614932<br>http://www.ncbi.nlm.nih.gov/pubmed/11274345<br>GADD45A = growth arrest and DNA-damage-inducible, alpha<br>GADD45B = growth arrest and DNA-damage-inducible, beta<br>GADD45G = growth arrest and DNA-damage-inducible, gamma<br>VARIABLE: GADD45 gene (any isoform) expression level<br>The GADD45 gene is inducible by various environmental stresses.<br>Smad-dependent gene expression can provoke p38 activation in response to TGFbeta. GADD45beta was identified as the TGFbeta-inducible gene whose expression activates the p38 pathway.<br>Expression of GADD45 genes in mammalian cells strongly activates co-expressed MTK1, as well as p38 and JNK, the MAPKs downstream of MTK1.<br>PMID:21614932<br>Expression of GADD45 is the culmination of a signaling pathway that requires prior expression of the tumor suppressor protein p53 which trans-activates the GADD45 gene.<br>PMID:11274345	
		**Regulator**	**Comment**
		SMAD	http://www.ncbi.nlm.nih.gov/pubmed/21614932<br>Smad-dependent gene expression can provoke p38 activation in response to TGFbeta. GADD45beta was identified as the TGFbeta-inducible gene whose expression activates the p38 pathway.<br>PMID:21614932
		p53	http://www.ncbi.nlm.nih.gov/pubmed/11274345<br>Expression of GADD45 is the culmination of a signaling pathway that requires prior expression of the tumor suppressor protein p53 which trans-activates the GADD45 gene.<br>PMID:11274345
GRB2	EGFR | FRS2 | TGFBR	http://www.genenames.org/data/hgnc_data.php?hgnc_id=4566<br>http://www.ncbi.nlm.nih.gov/pubmed/17496910	

		http://www.ncbi.nlm.nih.gov/pubmed/15567848<br>http://www.ncbi.nlm.nih.gov/pubmed/11997436<br>http://www.ncbi.nlm.nih.gov/pubmed/17673906<br>http://www.ncbi.nlm.nih.gov/pubmed/11447289<br>GRB2 = growth factor receptor-bound protein 2<br>VARIABLE: GRB2 recruitment by activated RTKs<br>Grb2 is an adaptor protein, normally present in cytosol. It is recruited to the plasma membrane by activated RTKs. Sos is recruited from the cytosol to the plasma membrane as a result of its constitutive interaction with Grb2. It is in an autoinhibited state.<br>PMID:17496910<br>The signaling pathways activated by FGFRs substantially overlap with those activated by EGFRs.<br>Grb2 molecules recruit the nucleotide exchange factor SOS, leading to the activation of the Ras-MAPK signaling cascade.<br>Grb2 recruits the docking protein Gab1, which is tyrosine phosphorylated by EGFR or FGFR, leading to recruitment and activation of the PI3K-Akt cell survival pathway.<br>The ubiquitin ligase Cbl interacts with EGFR directly and indirectly through Grb2, promoting ubiquitination and degradation of EGFR<br>Grb2, bound to Cbl, does not interact directly with FGFR but, rather, binds to tyrosine-phosphorylated FRS2, promoting ubiquitination and degradation of FRS2 and FGFR<br>PMID:15567848<br>FGF-induced tyrosine phosphorylation of FRS2 results in complex formation with the adaptor protein Grb2 bound to Cbl by means of its SH3 domains. FGF-induced ternary complex formation among FRS2, Grb2, and Cbl results in ubiquitination and degradation of FRS2 and FGF receptor (FGFR).<br>PMID:11997436<br>TGF-beta-induced ShcA phosphorylation induces ShcA association with Grb2 and Sos, thereby initiating the well-characterised pathway linking receptor tyrosine kinases with Erk MAP kinases.<br>PMID:17673906<br>FGF stimulation leads to phosphorylation of Shp2 on a tyrosine residue that forms a complex with an additional molecule of Grb2. Grb2/Sos complexes are thus recruited directly and indirectly via Shp2 upon tyrosine phosphorylation of FRS2a in response to growth factor stimulation.<br>PMID:11447289<br><br>**Regulator** — **Comment**<br>EGFR — http://www.ncbi.nlm.nih.gov/pubmed/17496910 Grb2 is an adaptor protein, normally present in cytosol. It is recruited to the plasma membrane by activated RTKs. PMID:17496910<br>TGFBR — http://www.ncbi.nlm.nih.gov/pubmed/17673906 TGF-beta-induced ShcA phosphorylation induces ShcA association with Grb2 and Sos, thereby initiating the well-characterised pathway linking receptor tyrosine

		kinases with Erk MAP kinases.<br>PMID:17673906<br>FRS2<br>http://www.ncbi.nlm.nih.gov/pubmed/11447289<br>FGF stimulation leads to phosphorylation of Shp2 on a tyrosine residue that forms a complex with an additional molecule of Grb2. Grb2/Sos complexes are thus recruited directly and indirectly via Shp2 upon tyrosine phosphorylation of FRS2a in response to growth factor stimulation.<br>PMID:11447289
Growth_Arrest	p21	http://www.ncbi.nlm.nih.gov/pubmed/8614832<br>Phenotype<br>VARIABLE: growth arrest enablement<br>Cell growth arrest is induced through cyclin-dependent kinase inhibitor p21 WAF1/CIP1.<br>PMID:8614832<br><br>**Regulator** / **Comment**<br>p21<br>http://www.ncbi.nlm.nih.gov/pubmed/8614832<br>Cell growth arrest is induced through cyclin-dependent kinase inhibitor p21 WAF1/CIP1.<br>PMID:8614832
JNK	(TAOK & MAP3K1_3) | (MAP3K1_3 & MTK1) | (TAOK & MTK1) | (TAK1 & MTK1) | (TAK1 & MAP3K1_3) | (TAK1 & TAOK) | ((TAOK | MTK1 | MAP3K1_3 | TAK1) & !DUSP1)	http://www.genenames.org/data/hgnc_data.php?hgnc_id=6881<br>http://www.genenames.org/data/hgnc_data.php?hgnc_id=6886<br>http://www.genenames.org/data/hgnc_data.php?hgnc_id=6872<br>http://www.ncbi.nlm.nih.gov/pubmed/21614932<br>http://www.ncbi.nlm.nih.gov/pubmed/19436832<br>http://www.ncbi.nlm.nih.gov/pubmed/20028971<br>http://www.ncbi.nlm.nih.gov/pubmed/11274345<br>http://www.ncbi.nlm.nih.gov/pubmed/20060931<br>MAPK8 = mitogen-activated protein kinase 8<br>MAPK9 = mitogen-activated protein kinase 9<br>MAPK10 = mitogen-activated protein kinase 10<br>VARIABLE: JNK (any isoform) phosphorylation level<br>p38 and JNK are activated after expression of GADD45, following stress, through MTK1.<br>Expression of GADD45 genes in mammalian cells strongly activates co-expressed MTK1, as well as p38 and JNK, the MAPKs downstream of MTK1.<br>TAO kinases are MAP3Ks that function upstream of p38 and JNK.<br>PMID:21614932<br>DUSP1 preferentially inactivates JNK and p38.<br>PMID:19436832

JNK phosphorylates MST1 at serine 82, which leads to the enhancement of MST1 activation. The activation of MST1 phosphorylates FOXO3 at serine 207 and promotes cell death.
PMID:20028971
JNK and p38 can both phosphorylate ATF2 at Thr69 and Ser71.
JNK can phosphorylate the c-Juntrans-activating domain at Ser63 and Ser73. JNKs, but not the ERKs or p38s, binds c-Jun quite strongly.
ELK1 is a nuclear JNK target.
MEKK1 can interact with Ras in a GTP-dependent manner. The GTP-dependent coupling of MEKK1 to Ras indicates that MEKK1 may be an effector for those agonists that recruit JNK through Ras-dependent mechanisms.
MEKK2 and MEKK3 can activate JNK, p38 and ERK pathways.
PMID:11274345
Activated TGFBR1/TGFBR2 complex attracts the TAB2/TAB3 proteins which promote activation of TAK1 (by phosphorylation), leading to activation of p38 and JNK.
PMID:20060931

**Regulator**	**Comment**
TAK1	http://www.ncbi.nlm.nih.gov/pubmed/20060931<br>Activated TGFBR1/TGFBR2 complex attracts the TAB2/TAB3 proteins which promote activation of TAK1 (by phosphorylation), leading to activation of p38 and JNK.<br>PMID:20060931
MAP3K1_3	http://www.ncbi.nlm.nih.gov/pubmed/11274345<br>The GTP-dependent coupling of MEKK1 to Ras indicates that MEKK1 may be an effector for those agonists that recruit JNK through Ras-dependent mechanisms. MEKK1 selectively activates the endogenous JNK pathway. MEKK1 can activate MEK4 and MEK7 in vivo.<br>MEKK2 and MEKK3 can activate JNK, p38 and ERK pathways.<br>PMID:11274345
DUSP1	http://www.ncbi.nlm.nih.gov/pubmed/19436832<br>DUSP1 preferentially inactivates JNK and p38.<br>PMID:19436832
TAOK	http://www.ncbi.nlm.nih.gov/pubmed/21614932<br>TAO kinases are MAP3Ks that function upstream of p38 and JNK.<br>PMID:21614932
MTK1	http://www.ncbi.nlm.nih.gov/pubmed/21614932<br>Expression of GADD45 genes in mammalian cells

		strongly activates co-expressed MTK1, as well as p38 and JNK, the MAPKs downstream of MTK1.<br>PMID:21614932
JUN	JNK	http://www.genenames.org/data/hgnc_data.php?hgnc_id=6204<br>http://www.ncbi.nlm.nih.gov/pubmed/11274345<br>JUN = jun proto-oncogene<br>VARIABLE: JUN phosphorylation level<br>AP-1 is a heterodimer comprised of bZIP transcription factors, typically c-Jun and JunD, along with members of the fos (usually c-Fos) and ATF (usually ATF2) families. All bZIP transcription factors contain leucine zippers that enable homo- and heterodimerisation, and AP-1 components are organised into Jun-Jun, Jun-Fos, or Jun-ATF dimers. JNK can phosphorylate the c-Jun trans-activating domain at Ser63 and Ser73. JNKs, but not the ERKs or p38s, binds c-Jun quite strongly.<br>PMID:11274345<br><br>**Regulator** / **Comment**<br>JNK / http://www.ncbi.nlm.nih.gov/pubmed/11274345<br>JNK can phosphorylate the c-Jun trans-activating domain at Ser63 and Ser73. JNKs, but not the ERKs or p38s, binds c-Jun quite strongly.<br>PMID:11274345
MAP3K1_3	RAS	http://www.genenames.org/data/hgnc_data.php?hgnc_id=6848<br>http://www.genenames.org/data/hgnc_data.php?hgnc_id=6854<br>http://www.genenames.org/data/hgnc_data.php?hgnc_id=6855<br>http://www.ncbi.nlm.nih.gov/pubmed/11274345<br>MAP3K1 = mitogen-activated protein kinase kinase kinase 1<br>MAP3K2 = mitogen-activated protein kinase kinase kinase 2<br>MAP3K3 = mitogen-activated protein kinase kinase kinase 3<br>VARIABLE: MAP3K1/2/3 (any isoform) phosphorylation level<br><br>The GTP-dependent coupling of MEKK1 to Ras indicates that MEKK1 may be an effector for those agonists that recruit JNK through Ras-dependent mechanisms. MEKK1 selectively activates the endogenous JNK pathway.<br>MEKK1 can activate MEK4 and MEK7 in vivo.<br>MEKK2 and MEKK3 can activate JNK, p38 and ERK pathways.<br>PMID:11274345<br><br>**Regulator** / **Comment**<br>RAS / http://www.ncbi.nlm.nih.gov/pubmed/11274345<br>The GTP-dependent coupling of MEKK1 to Ras indicates that MEKK1 may be an effector for those agonists that recruit JNK through Ras-dependent

		mechanisms. MEKK1 selectively activates the endogenous JNK pathway. MEKK1 can activate MEK4 and MEK7 in vivo.<br>MEKK2 and MEKK3 can activate JNK, p38 and ERK pathways.<br>PMID:11274345
MAX	p38	http://www.genenames.org/data/hgnc_data.php?hgnc_id=6913<br>http://www.ncbi.nlm.nih.gov/pubmed/11274345<br>MAX = MYC associated factor X<br>VARIABLE: MAX phosphorylation level<br>MAX interacts with the transcription factor c-Myc, enabling c-Myc to trans-activate at least a subset of its target genes.<br>MAX is phosphorylated (and activated) by p38, through complex formation.<br>PMID:11274345<br><br>**Regulator** — **Comment**<br>p38 — http://www.ncbi.nlm.nih.gov/pubmed/11274345<br>MAX is phosphorylated (and activated) by p38, through complex formation.<br>PMID:11274345
MDM2	(p53 | AKT) & !p14	http://www.genenames.org/data/hgnc_data.php?hgnc_id=6973<br>http://www.ncbi.nlm.nih.gov/pubmed/17158541<br>http://www.ncbi.nlm.nih.gov/pubmed/11850850<br>MDM2 = Mdm2, p53 E3 ubiquitin protein ligase homolog (mouse)<br>VARIABLE: MDM2 gene expression level<br>The Mdm2 protein is involved in an autoregulatory feedback loop with p53, thus controlling its activity. Increased p53 levels transactivate the MDM2 promoter causing its upregulation. The translated protein then binds to p53 and transports it to the proteasome for ubiquitin-mediated degradation. The resultant lowered p53 levels then reduce the levels of Mdm2. The MDM2 gene is, in turn, transcriptionally inhibited by p14, providing another fine level of control for p53 activity.<br>PMID:17158541<br>Effective recruitment of Akt by appropriate survival signals may lead to activation of Mdm2, inactivation of p53, and eventually inhibition of p53-dependent apoptosis.<br>PMID:11850850<br><br>**Regulator** — **Comment**<br>p53 — http://www.ncbi.nlm.nih.gov/pubmed/17158541<br>The Mdm2 protein is involved in an autoregulatory feedback loop with p53, thus controlling its activity. Increased p53 levels transactivate the MDM2 promoter

<table>
<tr><td></td><td></td><td>
<table>
<tr><td></td><td>causing its upregulation. The translated protein then binds to p53 and transports it to the proteasome for ubiquitin-mediated degradation. The resultant lowered p53 levels then reduce the levels of Mdm2.
PMID:17158541</td></tr>
<tr><td>p14</td><td>http://www.ncbi.nlm.nih.gov/pubmed/17158541
The MDM2 gene is transcriptionally inhibited by p14.
PMID:17158541</td></tr>
<tr><td>AKT</td><td>http://www.ncbi.nlm.nih.gov/pubmed/11882383
Effective recruitment of Akt by appropriate survival signals may lead to activation of Mdm2, inactivation of p53, and eventually inhibition of p53-dependent apoptosis.
PMID:11850850</td></tr>
</table>
</td></tr>
<tr><td>MEK1_2</td><td>RAF | MAP3K1_3) & !(PPP2CA | AP1)</td><td>
http://www.genenames.org/data/hgnc_data.php?hgnc_id=6840
http://www.genenames.org/data/hgnc_data.php?hgnc_id=6842
http://www.ncbi.nlm.nih.gov/pubmed/17496910
http://www.ncbi.nlm.nih.gov/pubmed/18039929
MAP2K1 = mitogen-activated protein kinase kinase 1
MAP2K2 = mitogen-activated protein kinase kinase 2
VARIABLE: MEK (any isoform) phosphorylation level
Once activated, Raf family members are capable of initiating the phosphorylation cascade, whereby Raf activates MEK, and MEK in turn activates ERK.
PMID:17496910
MEK is continuously dephosphorylated by PP2A (PPP2CA), whose activity is stimulated by p38: p38 activity increases the physical association between PP2A and MEK/ERK complex.
AP-1 mediated gene expression inhibits ERK phosphorylation.
PMID:18039929
<table>
<tr><td>Regulator</td><td>Comment</td></tr>
<tr><td>RAF</td><td>http://www.ncbi.nlm.nih.gov/pubmed/17496910
Once activated, Raf family members are capable of initiating the phosphorylation cascade, whereby Raf activates MEK, and MEK in turn activates ERK.
PMID:17496910</td></tr>
<tr><td>MAP3K1_3</td><td>http://www.ncbi.nlm.nih.gov/pubmed/11274345
MEKK2 and MEKK3 can activate JNK, p38 and ERK pathways.
PMID:11274345</td></tr>
<tr><td>AP1</td><td>http://www.ncbi.nlm.nih.gov/pubmed/18039929</td></tr>
</table>
</td></tr>
</table>

<table>
<tr><td></td><td></td><td><table><tr><td></td><td>AP-1 mediated gene expression inhibits ERK phosphorylation.
PMID:18039929</td></tr><tr><td>PPP2CA</td><td>http://www.ncbi.nlm.nih.gov/pubmed/18039929
MEK is continuously dephosphorylated by PP2A (PPP2CA), whose activity is stimulated by p38: p38 activity increases the physical association between PP2A and MEK/ERK complex.
PMID:18039929</td></tr></table></td></tr>
<tr><td>MSK</td><td>ERK | p38</td><td>http://www.genenames.org/data/hgnc_data.php?hgnc_id=10433
http://www.genenames.org/data/hgnc_data.php?hgnc_id=10434
http://www.ncbi.nlm.nih.gov/pubmed/15187187
http://www.ncbi.nlm.nih.gov/pubmed/17158541
RPS6KA4 = ribosomal protein S6 kinase, 90kDa, polypeptide 4
RPS6KA5 = ribosomal protein S6 kinase, 90kDa, polypeptide 5
VARIABLE: MSK (any isoform) phosphorylation level
MSK1 and MSK2 are potently activated (by phosphorylation) in vivo by ERK1/2 and p38 but not JNK.
MSK1 mediates the mitogen-stimulated phoshphorylation of CREB.
PMID:15187187
Translocation of ERK into the nucleus activates the mitogen activated and stress-activated protein kinase 1 (MSK1), a histone H3 kinase that can relax chromatin, thus making it more transcriptionally accessible. This alteration in the chromatin state induces MYC, a gene that encodes the c-Myc protein, a transcription factor that controls the cell cycle.
PMID:17158541

<table><tr><th>Regulator</th><th>Comment</th></tr><tr><td>ERK</td><td>http://www.ncbi.nlm.nih.gov/pubmed/15187187
MSK1 and MSK2 are potently activated (by phosphorylation) in vivo by ERK1/2 and p38 but not JNK.
PMID:15187187</td></tr><tr><td>p38</td><td>http://www.ncbi.nlm.nih.gov/pubmed/15187187
MSK1 and MSK2 are potently activated (by phosphorylation) in vivo by ERK1/2 and p38 but not JNK.
PMID:15187187</td></tr></table></td></tr>
<tr><td>MTK1</td><td>GADD45</td><td>http://www.genenames.org/data/hgnc_data.php?hgnc_id=6856
http://www.ncbi.nlm.nih.gov/pubmed/21614932
MAP3K4 = mitogen-activated protein kinase kinase kinase 4
VARIABLE: MTK1 phosphorylation level</td></tr>
</table>

		The delayed activation of p38 by TGFbeta is mediated mainly by Smad-dependent GADD45beta expression and by its subsequent activation of MTK1.<br>Expression of GADD45 genes in mammalian cells strongly activates co-expressed MTK1, as well as p38 and JNK, the MAPKs downstream of MTK1.<br>PMID:21614932<br><br>**Regulator** / **Comment**<br>GADD45 / http://www.ncbi.nlm.nih.gov/pubmed/21614932<br>Expression of GADD45 genes in mammalian cells strongly activates co-expressed MTK1, as well as p38 and JNK, the MAPKs downstream of MTK1.<br>PMID:21614932
MYC	(MSK & MAX) | (MSK & AKT)	http://www.genenames.org/data/hgnc_data.php?hgnc_id=7553<br>http://www.ncbi.nlm.nih.gov/pubmed/17158541<br>http://www.ncbi.nlm.nih.gov/pubmed/11274345<br>http://www.ncbi.nlm.nih.gov/pubmed/12040186<br>http://www.ncbi.nlm.nih.gov/pubmed/11805123<br>MYC = v-myc myelocytomatosis viral oncogene homolog (avian)<br>VARIABLE: MYC activation level (implies gene expression)<br>Modelling hypotheses: MSK determines MYC gene expression, so it is always needed for MYC activation; MAX activates MYC protein; AKT inhibits GSK3B, which in turn inactivates MYC protein<br>Translocation of ERK into the nucleus activates the mitogen activated and stress-activated protein kinase 1 (MSK1), a histone H3 kinase that can relax chromatin, thus making it more transcriptionally accessible. This alteration in the chromatin state induces MYC, a gene that encodes the c-Myc protein, a transcription factor that controls the cell cycle. c-Myc signals downstream to promote expression of cyclins that complex with CDKs.<br>MYC inhibits p16, whereas it activates p14.<br>PMID:17158541<br>MAX interacts with the transcription factor c-Myc (this interaction is modelled here by complex formation) enabling c-Myc to trans-activate at least a subset of its target genes.<br>PMID:11274345<br>Glycogen synthase kinase 3 (GSK3) is a target of Akt. This protein kinase is constitutively active in unstimulated cells and phosphorylates many proteins (including glycogen synthase, c-Myc, and cyclin D) to keep them in inactive states or promote their degradation. Phosphorylation of GSK3 (both alpha and beta isoforms) by Akt turns off the catalytic activity of this enzyme, resulting in the activation of pathways that are normally repressed by GSK3.<br>PMID:12040186<br>MYC contributes to E2F-induced cell cycle progression.<br>PMID:11805123<br><br>**Regulator** / **Comment**<br>MSK / http://www.ncbi.nlm.nih.gov/pubmed/17158541

		Translocation of ERK into the nucleus activates the mitogen activated and stress-activated protein kinase 1 (MSK1), a histone H3 kinase that can relax chromatin, thus making it more transcriptionally accessible. This alteration in the chromatin state induces MYC, a gene that encodes the c-Myc protein, a transcription factor that controls the cell cycle.<br>PMID:17158541<br>MAX — http://www.ncbi.nlm.nih.gov/pubmed/11274345<br>MAX interacts with the transcription factor c-Myc, enabling c-Myc to trans-activate at least a subset of its target genes.<br>PMID:11274345<br>AKT — http://www.ncbi.nlm.nih.gov/pubmed/12040186<br>Glycogen synthase kinase 3 (GSK3) is a target of Akt. This protein kinase is constitutively active in unstimulated cells and phosphorylates many proteins (including glycogen synthase, c-Myc, and cyclin D) to keep them in inactive states or promote their degradation. Phosphorylation of GSK3 (both alpha and beta isoforms) by Akt turns off the catalytic activity of this enzyme, resulting in the activation of pathways that are normally repressed by GSK3.<br>PMID:12040186
PDK1	PI3K	http://www.genenames.org/data/hgnc_data.php?hgnc_id=8809<br>http://www.ncbi.nlm.nih.gov/pubmed/12040186<br>PDK1 = pyruvate dehydrogenase kinase, isozyme 1<br>VARIABLE: PDK1 activation level<br>PDK1 is a PI3K target, leading to activation of p70 and subsequent cell growth.<br>Signaling proteins with pleckstrin-homology (PH) domains accumulate at sites of PI3K activation by directly binding to PI(3,4,5)P3. Of particular interest are the protein serine-threonine kinases Akt and phosphoinositide-dependent kinase 1 (PDK1). Association with PI(3,4,5)P3 at the membrane brings these proteins into proximity and facilitates phosphorylation of Akt by PDK1. This phosphorylation stimulates the catalytic activity of Akt, resulting in the phosphorylation of a host of other proteins that affect cell growth, cell cycle entry, and cell survival.<br>PMID:12040186<br><br>**Regulator** — **Comment**<br>PI3K — http://www.ncbi.nlm.nih.gov/pubmed/15199124<br>Phosphatidylinositol-3,4,5-triphosphate (PIP3), the reaction product of PI 3-kinase, is responsible for

		activation of phosphoinositide-dependent kinase (PDK) and the antiapoptotic protein kinase Akt.<br>PMID:15199124
PI3K	GAB1 | (RAS & SOS)	http://www.genenames.org/data/hgnc_data.php?hgnc_id=8975<br>http://www.ncbi.nlm.nih.gov/pubmed/15567848<br>http://www.ncbi.nlm.nih.gov/pubmed/15199124<br>http://www.ncbi.nlm.nih.gov/pubmed/21779497<br>PIK3CA = phosphoinositide-3-kinase, catalytic, alpha polypeptide<br>VARIABLE: PI3K activation level<br>Grb2 recruits the docking protein Gab1, which is tyrosine phosphorylated by FGFR, leading to recruitment and activation of the PI3K-Akt cell survival pathway.<br>EGF stimulates a cell survival pathway mediated by phosphoinositide 3-kinase (PI3K) and the protein kinase Akt by an indirect mechanism in which tyrosine phosphorylation of the docking protein Gab1 or ErbB3 by EGFR leads to recruitment and activation of PI3K.<br>PMID:15567848<br>Recruitment and activation of PI 3-kinase by Gab1 results in a positive-feedback loop mediated by binding of the PH domain of Gab1 to the product of PI3-kinase activation, phosphatidylinositol-3,4,5-triphosphate.<br>Phosphatidylinositol-3,4,5-triphosphate (PIP3), the reaction product of PI 3-kinase, is responsible for activation of phosphoinositide-dependent kinase (PDK) and the antiapoptotic protein kinase Akt.<br>PMID:15199124<br>PI3K is a well characterised effector of RAS, through GRB2/SOS pathway.<br>PMID:21779497<br><br>**Regulator** — **Comment**<br>RAS — http://www.ncbi.nlm.nih.gov/pubmed/21779497<br>PI3K is a well characterised effector of RAS, through GRB2/SOS pathway.<br>PMID:21779497<br>SOS — http://www.ncbi.nlm.nih.gov/pubmed/21779497<br>PI3K is a well characterised effector of RAS, through GRB2/SOS pathway.<br>PMID:21779497<br>GAB1 — http://www.ncbi.nlm.nih.gov/pubmed/15199124<br>Recruitment of PI 3-kinase by Gab1 results in a positive-feedback loop mediated by binding of the PH domain of Gab1 to the product of PI3-kinase activation, phosphatidylinositol-3,4,5-triphosphate.<br>PMID:15199124
PKC	PLCG	http://www.genenames.org/data/hgnc_data.php?hgnc_id=9393<br>http://www.genenames.org/data/hgnc_data.php?hgnc_id=9395

<table>
<tr><td></td><td></td><td>http://www.genenames.org/data/hgnc_data.php?hgnc_id=9402
http://www.ncbi.nlm.nih.gov/pubmed/15567848
http://www.ncbi.nlm.nih.gov/pubmed/6321473
http://www.ncbi.nlm.nih.gov/pubmed/8321321
http://www.ncbi.nlm.nih.gov/pubmed/9694798
http://www.ncbi.nlm.nih.gov/pubmed/8622701
http://www.ncbi.nlm.nih.gov/pubmed/14699054
PRKCA = protein kinase C, alpha
PRKCB = protein kinase C, beta
PRKCG = protein kinase C, gamma
VARIABLE: PKC (any isoform) enzymatic activity
Recruitment to the membrane and tyrosine phosphorylation enhance the enzymatic activity of PLC-g, leading to the formation of two second messengers, diacylglycerol (DAG) and inositol 1,4,5-trisphosphate (IP3). IP3 releases Ca2+ from internal stores, which in turn acts in concert with DAG to translocate protein kinase C (PKC) to the cell membrane and stimulate its enzymatic activity.
PMID:15567848
PKC may cause phosphorylation of EGFR, leading to a decrease in its activity.
PMID:6321473
PKCalpha can directly phosphorylate and activates Raf-1.
PMID:8321321
(Inferred from FGFR1) PKC-mediated phosphorylation can lead to internalization and degradation of FGFR1.
PMID:9694798, PMID:8622701, PMID:14699054

<table><tr><th>Regulator</th><th>Comment</th></tr><tr><td>PLCG</td><td>http://www.ncbi.nlm.nih.gov/pubmed/15567848
Recruitment to the membrane and tyrosine phosphorylation enhance the enzymatic activity of PLC-g, leading to the formation of two second messengers, diacylglycerol (DAG) and inositol 1,4,5-trisphosphate (IP3). IP3 releases Ca2+ from internal stores, which in turn acts in concert with DAG to translocate protein kinase C (PKC) to the cell membrane and stimulate its enzymatic activity.
PMID:15567848</td></tr></table></td></tr>
<tr><td>PLCG</td><td>EGFR | FGFR3</td><td>http://www.genenames.org/data/hgnc_data.php?hgnc_id=9065
http://www.genenames.org/data/hgnc_data.php?hgnc_id=9066
http://www.ncbi.nlm.nih.gov/pubmed/1656221
http://www.ncbi.nlm.nih.gov/pubmed/2472219
http://www.ncbi.nlm.nih.gov/pubmed/15567848
http://www.ncbi.nlm.nih.gov/pubmed/17496910</td></tr>
</table>

<table>
<tr><td></td><td></td><td>http://www.ncbi.nlm.nih.gov/pubmed/16488589
PLCG1 = phospholipase C, gamma 1
PLCG2 = phospholipase C, gamma 2 (phosphatidylinositol-specific)
VARIABLE: PLCG (any isoform) phosphorylation level
Binding of FGF to FGFR leads to tyrosine phosphorylation of PLCgamma.
PMID:1656221
Tyrosine phosphorylation of PLC-gamma by EGF receptor leads to its activation.
PMID:2472219
Recruitment to the membrane and tyrosine phosphorylation enhance the enzymatic activity of PLC-g, leading to the formation of two second messengers, diacylglycerol (DAG) and inositol 1,4,5-trisphosphate (IP3). IP3 releases Ca2+ from internal stores, which in turn acts in concert with DAG to translocate protein kinase C (PKC) to the cell membrane and stimulate its enzymatic activity.
PMID:15567848
RasGRP1 is a C1-domain containing protein that is activated by DAG and Ca2+, in a manner analogous to members of the PKC family.
PMID:17496910
Calcium liberated from internal stores by IP3 acts on the calcium- and DAG-sensitive RasGRP1 and causes it to translocate to the Golgi. RasGRP1 activates Golgi-associated Ras on this compartment.
PMID:16488589
<table>
<tr><th>Regulator</th><th>Comment</th></tr>
<tr><td>EGFR</td><td>http://www.ncbi.nlm.nih.gov/pubmed/2472219
Tyrosine phosphorylation of PLC-gamma by EGF receptor leads to its activation.
PMID:2472219</td></tr>
<tr><td>FGFR3</td><td>http://www.ncbi.nlm.nih.gov/pubmed/1656221
Binding of FGF to FGFR leads to tyrosine phosphorylation of PLCgamma.
PMID:1656221</td></tr>
</table></td></tr>
<tr><td>PPP2CA</td><td>p38</td><td>http://www.genenames.org/data/hgnc_data.php?hgnc_id=9299
http://www.ncbi.nlm.nih.gov/pubmed/18039929
PPP2CA = protein phosphatase 2, catalytic subunit, alpha isozyme
VARIABLE: PPP2CA activation level
MEK is continuously dephosphorylated by PP2A (PPP2CA), whose activity is stimulated by p38: p38 activity increases the physical association between PP2A and MEK/ERK complex.
PMID:18039929
<table>
<tr><th>Regulator</th><th>Comment</th></tr>
<tr><td>p38</td><td>http://www.ncbi.nlm.nih.gov/pubmed/18039929
MEK is continuously dephosphorylated by PP2A</td></tr>
</table></td></tr>
</table>

		(PPP2CA), whose activity is stimulated by p38: p38 activity increases the physical association between PP2A and MEK/ERK complex.<br>PMID:18039929
Proliferation	p70 & MYC & !p21	http://www.ncbi.nlm.nih.gov/pubmed/12040186<br>http://www.ncbi.nlm.nih.gov/pubmed/11805123<br>http://www.ncbi.nlm.nih.gov/pubmed/8614832<br>Proliferation<br>VARIABLE: proliferation enablement<br>Proliferation is an output variable of the model.<br>Modelling hypotheses: proliferation is supposed to be enabled when both MYC and p70 are activated, and p21 (CDK inhibitor) is inactive<br>PDK1 is a PI3K target, leading to activation of p70 and subsequent cell growth.<br>PMID:12040186<br>MYC contributes to E2F-induced cell cycle progression.<br>PMID:11805123<br>Cell growth arrest is induced through cyclin-dependent kinase inhibitor p21 WAF1/CIP1.<br>PMID:8614832<br><br>**Regulator** — **Comment**<br>p70 — http://www.ncbi.nlm.nih.gov/pubmed/12040186 PDK1 is a PI3K target, leading to activation of p70 and subsequent cell growth. PMID:12040186<br>MYC — http://www.ncbi.nlm.nih.gov/pubmed/11805123 MYC contributes to E2F-induced cell cycle progression. PMID:11805123<br>p21 — http://www.ncbi.nlm.nih.gov/pubmed/8614832 Cell growth arrest is induced through cyclin-dependent kinase inhibitor p21 WAF1/CIP1. PMID:8614832
PTEN	p53	http://www.genenames.org/data/hgnc_data.php?hgnc_id=9588<br>http://www.ncbi.nlm.nih.gov/pubmed/12217521<br>PTEN = phosphatase and tensin homolog<br>VARIABLE: PTEN protein expression level<br>p53 induces transcription of the PTEN gene and elevates cellular levels of the PTEN protein, which in turn inhibits activation of Akt.<br>PMID:12217521<br><br>**Regulator** — **Comment**

		p53 http://www.ncbi.nlm.nih.gov/pubmed/12217521<br>p53 induces transcription of the PTEN gene and elevates cellular levels of the PTEN protein, which in turn inhibits activation of Akt.<br>PMID:12217521
p14	MYC	http://www.genenames.org/data/hgnc_data.php?hgnc_id=1787<br>http://www.ncbi.nlm.nih.gov/pubmed/17158541<br>CDKN2A = cyclin-dependent kinase inhibitor 2A (melanoma, p16, inhibits CDK4)<br>VARIABLE: p14 activation level<br>MYC inhibits p16, whereas it activates p14.<br>The MDM2 gene is transcriptionally inhibited by p14.<br>PMID:17158541<br><br>**Regulator** — **Comment**<br>MYC — http://www.ncbi.nlm.nih.gov/pubmed/17158541<br>MYC inhibits p16, whereas it activates p14.<br>PMID:17158541
p21	!AKT & p53	http://www.genenames.org/data/hgnc_data.php?hgnc_id=1784<br>http://www.ncbi.nlm.nih.gov/pubmed/11882383<br>http://www.ncbi.nlm.nih.gov/pubmed/17158541<br>http://www.ncbi.nlm.nih.gov/pubmed/8614832<br>CDKN1A = cyclin-dependent kinase inhibitor 1A (p21, Cip1)<br>VARIABLE: p21 activation level<br>p21 is a direct substrate of AKT and this may regulate the subcellular localisation of p21. Phosphorylation of p21 by AKT correlated with its exit from the nucleus and with increased cell cycle progression.<br>PMID:11882383<br><br>p21 is transcriptionally induced by the p53 protein.<br>p21 is a CDK inhibitor.<br>PMID:17158541<br>Cell growth arrest is induced through cyclin-dependent kinase inhibitor p21 WAF1/CIP1.<br>PMID:8614832<br><br>**Regulator** — **Comment**<br>p53 — http://www.ncbi.nlm.nih.gov/pubmed/17158541<br>p21 is transcriptionally induced by the p53 protein.<br>PMID:17158541<br>AKT — http://www.ncbi.nlm.nih.gov/pubmed/11882383<br>p21 is a direct substrate of AKT and this may regulate the subcellular localisation of p21. Phosphorylation of p21

		by AKT correlated with its exit from the nucleus and with increased cell cycle progression.<br>PMID:11882383
p38	(TAOK & MAP3K1_3) | (MAP3K1_3 & MTK1) | (TAOK & MTK1) | (TAK1 & MTK1) | (TAK1 & MAP3K1_3) | (TAK1 & TAOK) | ((TAOK | MTK1 | MAP3K1_3 | TAK1) & !DUSP1)	http://www.genenames.org/data/hgnc_data.php?hgnc_id=6873<br>http://www.genenames.org/data/hgnc_data.php?hgnc_id=6874<br>http://www.genenames.org/data/hgnc_data.php?hgnc_id=6875<br>http://www.genenames.org/data/hgnc_data.php?hgnc_id=6876<br>http://www.ncbi.nlm.nih.gov/pubmed/21614932<br>http://www.ncbi.nlm.nih.gov/pubmed/19436832<br>http://www.ncbi.nlm.nih.gov/pubmed/11274345<br>http://www.ncbi.nlm.nih.gov/pubmed/15187187<br>http://www.ncbi.nlm.nih.gov/pubmed/20506250<br>http://www.ncbi.nlm.nih.gov/pubmed/18039929<br>http://www.ncbi.nlm.nih.gov/pubmed/20060931<br>MAPK11 = mitogen-activated protein kinase 11<br>MAPK12 = mitogen-activated protein kinase 12<br>MAPK13 = mitogen-activated protein kinase 13<br>MAPK14 = mitogen-activated protein kinase 14<br>VARIABLE: p38 (any isoform) phosphorylation level<br>p38 and JNK are activated after expression of GADD45, following stress, through MTK1.<br>Expression of GADD45 genes in mammalian cells strongly activates co-expressed MTK1, as well as p38 and JNK, the MAPKs downstream of MTK1.<br>TAO kinases are MAP3Ks that function upstream of p38 and JNK.<br>Activated p38 phosphorylates p53 at several residues, including Ser33, and thereby increases the transcriptional activity of p53. Activated p53 then induces apoptosis in cells that have suffered extensive DNA damage. When the intensity of the damage is moderate, however, the cell can repair the damage and escape apoptotic cell death by down-regulating p38-p53 signaling. Under these conditions, p53-induced Wip1 phosphatase interacts with activated p38 and selectively dephosphorylates p38 at the phospho-Thr180 residue, thereby reducing its kinase activity towards p53.<br>PMID:21614932<br>DUSP1 preferentially inactivates JNK and p38.<br>PMID:19436832<br>JNK and p38 can both phosphorylate ATF2 at Thr69 and Ser71.<br>MAX is phosphorylated (and activated) by p38, through complex formation.<br>MEKK2 and MEKK3 can activate JNK, p38 and ERK pathways.<br>PMID:11274345<br>MSK1 and MSK2 are potently activated (by phosphorylation) in vivo by ERK1/2 and p38 but not JNK.<br>PMID:15187187<br>ELK1 is a nuclear p38 target.<br>PMID:20506250

<table>
<tr><td></td><td></td><td>MEK is continuously dephosphorylated by PP2A (PPP2CA), whose activity is stimulated by p38: p38 activity increases the physical association between PP2A and MEK/ERK complex.
PMID:18039929
Activated TGFBR1/TGFBR2 complex attracts the TAB2/TAB3 proteins which promote activation of TAK1 (by phosphorylation), leading to activation of p38 and JNK.
PMID:20060931

<table>
<tr><th>Regulator</th><th>Comment</th></tr>
<tr><td>TAK1</td><td>http://www.ncbi.nlm.nih.gov/pubmed/20060931
Activated TGFBR1/TGFBR2 complex attracts the TAB2/TAB3 proteins which promote activation of TAK1 (by phosphorylation), leading to activation of p38 and JNK.
PMID:20060931</td></tr>
<tr><td>MAP3K1_3</td><td>http://www.ncbi.nlm.nih.gov/pubmed/11274345
MEKK2 and MEKK3 can activate JNK, p38 and ERK pathways.
PMID:11274345</td></tr>
<tr><td>DUSP1</td><td>http://www.ncbi.nlm.nih.gov/pubmed/19436832
DUSP1 preferentially inactivates JNK and p38.
PMID:19436832</td></tr>
<tr><td>TAOK</td><td>http://www.ncbi.nlm.nih.gov/pubmed/21614932
TAO kinases are MAP3Ks that function upstream of p38 and JNK.
PMID:21614932</td></tr>
<tr><td>MTK1</td><td>http://www.ncbi.nlm.nih.gov/pubmed/21614932
Expression of GADD45 genes in mammalian cells strongly activates co-expressed MTK1, as well as p38 and JNK, the MAPKs downstream of MTK1.
PMID:21614932</td></tr>
</table>
</td></tr>
<tr><td>p53</td><td>(ATM & p38) | ((ATM | p38) & ! MDM2)</td><td>http://www.genenames.org/data/hgnc_data.php?hgnc_id=11998
http://www.ncbi.nlm.nih.gov/pubmed/15140942
http://www.ncbi.nlm.nih.gov/pubmed/21614932
http://www.ncbi.nlm.nih.gov/pubmed/17158541
http://www.ncbi.nlm.nih.gov/pubmed/11274345
http://www.ncbi.nlm.nih.gov/pubmed/19641508
http://www.ncbi.nlm.nih.gov/pubmed/12217521
TP53 = tumor protein p53
VARIABLE: p53 phosphorylation level
ATM phosphorylates p53 at ser15 and stabilize it.</td></tr>
</table>

PMID:15140942
Activated p38 phosphorylates p53 at several residues, including Ser33, and thereby increases the transcriptional activity of p53. Activated p53 then induces apoptosis in cells that have suffered extensive DNA damage. When the intensity of the damage is moderate, however, the cell can repair the damage and escape apoptotic cell death by down-regulating p38-p53 signaling. Under these conditions, p53-induced Wip1 phosphatase interacts with activated p38 and selectively dephosphorylates p38 at the phospho-Thr180 residue, thereby reducing its kinase activity towards p53.
PMID:21614932
The Mdm2 protein is involved in an autoregulatory feedback loop with p53, thus controlling its activity. Increased p53 levels transactivate the MDM2 promoter causing its upregulation. The translated protein then binds to p53 and transports it to the proteasome for ubiquitin-mediated degradation. The resultant lowered p53 levels then reduce the levels of Mdm2. p21 is transcriptionally regulated by the p53 protein.
PMID:17158541
Expression of GADD45 is the culmination of a signaling pathway that requires prior expression of the tumor suppressor protein p53 which trans-activates the GADD45 gene.
PMID:11274345
In response to genotoxic stress such as DNA damage, PUMA is transactivated by p53 (leading to apoptosis).
PMID:19641508
p53 induces transcription of the PTEN gene and elevates cellular levels of the PTEN protein.
PMID:12217521

**Regulator**	**Comment**
ATM	http://www.ncbi.nlm.nih.gov/pubmed/15140942 ATM phosphorylates p53 at ser15 and stabilize it. PMID:15140942
p38	http://www.ncbi.nlm.nih.gov/pubmed/21614932 Activated p38 phosphorylates p53 at several residues, including Ser33, and thereby increases the transcriptional activity of p53. Activated p53 then induces apoptosis in cells that have suffered extensive DNA damage. When the intensity of the damage is moderate, however, the cell can repair the damage and escape apoptotic cell death by down-regulating p38-p53 signaling. Under these conditions, p53-induced Wip1 phosphatase interacts with activated p38 and selectively dephosphorylates p38 at the phospho-Thr180 residue, thereby reducing its kinase activity towards p53. PMID:21614932
MDM2	http://www.ncbi.nlm.nih.gov/pubmed/17158541 The Mdm2 protein is involved in an autoregulatory feedback loop with p53, thus controlling its activity.

		Increased p53 levels transactivate the MDM2 promoter causing its upregulation. The translated protein then binds to p53 and transports it to the proteasome for ubiquitin-mediated degradation. The resultant lowered p53 levels then reduce the levels of Mdm2.<br>PMID:17158541
p70	PDK1 & ERK	http://www.genenames.org/data/hgnc_data.php?hgnc_id=10436<br>http://www.genenames.org/data/hgnc_data.php?hgnc_id=10437<br>http://www.ncbi.nlm.nih.gov/pubmed/12040186<br>http://www.ncbi.nlm.nih.gov/pubmed/11940578<br>http://www.ncbi.nlm.nih.gov/pubmed/10601235<br>http://www.ncbi.nlm.nih.gov/pubmed/11431469<br>RPS6KB1 = ribosomal protein S6 kinase, 70kDa, polypeptide 1<br>RPS6KB2 = ribosomal protein S6 kinase, 70kDa, polypeptide 2<br>VARIABLE: p70 (any isoform) phosphorylation level<br>PDK1 is a PI3K target, leading to activation of p70 (through phosphorylation) and subsequent cell growth.<br>PMID:12040186<br>p70 activation requires both ERK cascade and PI3K/AKT cascade, at least in some cell types.<br>PMID:11940578, PMID:10601235, PMID:11431469<br><br>**Regulator** — **Comment**<br>ERK — http://www.ncbi.nlm.nih.gov/pubmed/11940578<br>http://www.ncbi.nlm.nih.gov/pubmed/10601235<br>http://www.ncbi.nlm.nih.gov/pubmed/11431469<br>p70 activation requires both ERK cascade and PI3K/AKT cascade, at least in some cell types.<br>PMID:11940578, PMID:10601235, PMID:11431469<br>PDK1 — http://www.ncbi.nlm.nih.gov/pubmed/12040186<br>PDK1 is a PI3K target, leading to activation of p70 and subsequent cell growth.<br>PMID:12040186
RAF	(RAS | PKC) & !(ERK | AKT)	http://www.genenames.org/data/hgnc_data.php?hgnc_id=9829<br>http://www.genenames.org/data/hgnc_data.php?hgnc_id=1097<br>http://www.genenames.org/data/hgnc_data.php?hgnc_id=646<br>http://www.ncbi.nlm.nih.gov/pubmed/17496910<br>http://www.ncbi.nlm.nih.gov/pubmed/8321321<br>http://www.ncbi.nlm.nih.gov/pubmed/18039929<br>http://www.ncbi.nlm.nih.gov/pubmed/19933846<br>RAF1 = v-raf-1 murine leukemia viral oncogene homolog 1<br>BRAF = v-raf murine sarcoma viral oncogene homolog B1

<table>
<tr><td></td><td></td><td>ARAF = v-raf murine sarcoma 3611 viral oncogene homolog
VARIABLE: RAF (any isoform) phosphorylation
Once activated, Raf family members are capable of initiating the phosphorylation cascade, whereby Raf activates MEK, and MEK in turn activates ERK.
PMID:17496910
PKCalpha directly phosphorylates and activates Raf-1.
PMID:8321321
Activated Ras recruits cytoplasmic Raf (MAPKKK) for activation. There are three mammalian serine/threonine Raf kinases: A-Raf, B-Raf, and Raf-1 (also known as C-Raf). All three Raf proteins share the same downstream MAPKK substrate mitogen activated protein kinase kinases 1,2 (MEK1,2).
PMID:18039929
Phosphorylation by Akt inhibits EGF-dependent activity of B-Raf and C-Raf.
PMID:10869359
B-Raf phosphorylated by activated ERK and find that feedback phosphorylation of B-Raf inhibits binding to activated Ras.
PMID:19933846
<table>
<tr><th>Regulator</th><th>Comment</th></tr>
<tr><td>ERK</td><td>http://www.ncbi.nlm.nih.gov/pubmed/19933846
B-Raf phosphorylated by activated ERK and find that feedback phosphorylation of B-Raf inhibits binding to activated Ras.
PMID:19933846</td></tr>
<tr><td>RAS</td><td>http://www.ncbi.nlm.nih.gov/pubmed/18039929
Activated Ras recruits cytoplasmic Raf (MAPKKK) for activation (through phosphorylation). There are three mammalian serine/threonine Raf kinases: A-Raf, B-Raf, and Raf-1 (also known as C-Raf). All three Raf proteins share the same downstream MAPKK substrate mitogen activated protein kinase kinases 1,2 (MEK1,2).
PMID:18039929</td></tr>
<tr><td>AKT</td><td>http://www.ncbi.nlm.nih.gov/pubmed/10869359
Phosphorylation by Akt inhibits EGF-dependent activity of B-Raf and C-Raf.
PMID:10869359</td></tr>
<tr><td>PKC</td><td>http://www.ncbi.nlm.nih.gov/pubmed/8321321
PKCalpha can directly phosphorylate and activates Raf-1.
PMID:8321321</td></tr>
</table></td></tr>
<tr><td>RAS</td><td>SOS | PLCG</td><td>http://www.genenames.org/data/hgnc_data.php?hgnc_id=5173
http://www.genenames.org/data/hgnc_data.php?hgnc_id=6407</td></tr>
</table>

http://www.genenames.org/data/hgnc_data.php?hgnc_id=7989
http://www.ncbi.nlm.nih.gov/pubmed/18039929
http://www.ncbi.nlm.nih.gov/pubmed/11274345
http://www.ncbi.nlm.nih.gov/pubmed/21779497
http://www.ncbi.nlm.nih.gov/pubmed/16488589
HRAS = v-Ha-ras Harvey rat sarcoma viral oncogene homolog
KRAS = v-Ki-ras2 Kirsten rat sarcoma viral oncogene homolog
NRAS = neuroblastoma RAS viral (v-ras) oncogene homolog
VARIABLE: RAS (any isoform) phosphorylation level
Grb2 molecules recruit the nucleotide exchange factor SOS, leading to the activation of the Ras-MAPK signaling cascade.
Most of the signals activating the ERK pathway are initiated through receptor-mediated activation of the small G-protein, Ras. Ras is a membrane-bound protein activated through the exchange of bound GDP to GTP. The process of activating Ras thereby requires the recruitment of proteins responsible for initiating GDP/GTP exchange to the membrane, such as SOS (son of sevenless).
Activated Ras recruits cytoplasmic Raf (MAPKKK) for activation (through phosphorylation). There are three mammalian serine/threonine Raf kinases: A-Raf, B-Raf, and Raf-1 (also known as C-Raf). All three Raf proteins share the same downstream MAPKK substrate mitogen activated protein kinase kinases 1,2 (MEK1,2).
PMID:18039929
The GTP-dependent coupling of MEKK1 to Ras indicates that MEKK1 may be an effector for those agonists that recruit JNK through Ras-dependent mechanisms. MEKK1 selectively activates the endogenous JNK pathway.
MEKK1 can activate MEK4 and MEK7 in vivo.
MEKK2 and MEKK3 can activate JNK, p38 and ERK pathways.
PMID:11274345
PI3K is a well characterised effector of RAS, through GRB2/SOS pathway.
PMID:21779497
Calcium liberated from internal stores by IP3 acts on the calcium- and DAG-sensitive RasGRP1 and causes it to translocate to the Golgi. RasGRP1 activates Golgi-associated Ras on this compartment.
PMID:16488589

Regulator	Comment
SOS	http://www.ncbi.nlm.nih.gov/pubmed/18039929<br>The process of activating Ras requires the recruitment of proteins responsible for initiating GDP/GTP exchange to the membrane, such as SOS (son of sevenless).<br>PMID:18039929
PLCG	http://www.ncbi.nlm.nih.gov/pubmed/17496910<br>http://www.ncbi.nlm.nih.gov/pubmed/16488589<br>RasGRP1 is a C1-domain containing protein that is activated by DAG and Ca2+, in a manner analogous to members of the PKC family.

		PMID:17496910<br>Calcium liberated from internal stores by IP3 acts on the calcium- and DAG-sensitive RasGRP1 and causes it to translocate to the Golgi. RasGRP1 activates Golgi-associated Ras on this compartment.<br>PMID:16488589
RSK	ERK	http://www.genenames.org/data/hgnc_data.php?hgnc_id=10430<br>http://www.genenames.org/data/hgnc_data.php?hgnc_id=10431<br>http://www.genenames.org/data/hgnc_data.php?hgnc_id=10432<br>http://www.genenames.org/data/hgnc_data.php?hgnc_id=10435<br>http://www.ncbi.nlm.nih.gov/pubmed/15239952<br>http://www.ncbi.nlm.nih.gov/pubmed/16393692<br>http://www.ncbi.nlm.nih.gov/pubmed/9242373<br>RPS6KA1 = ribosomal protein S6 kinase, 90kDa, polypeptide 1<br>RPS6KA2 = ribosomal protein S6 kinase, 90kDa, polypeptide 2<br>RPS6KA3 = ribosomal protein S6 kinase, 90kDa, polypeptide 3<br>RPS6KA6 = ribosomal protein S6 kinase, 90kDa, polypeptide 6<br>VARIABLE: RSK (any isoform) phosphorylation level<br>RSK2 is a well known ERK substrate in the cytoplasm and has been shown to undergo autophosphorylation after ERK phosphorylation.<br>PMID:15239952<br>Phosphorylation by both ERKs and their downstream RSKs can stabilise the c-Fos protein for several hours. The combination of these phosphorylations allows c-Fos sustained activity.<br>PMID:16393692<br>p90 Rsk-2 is involved in SOS phosphorylation and may be important in down-regulation of the growth factor response.<br>PMID:9242373<br><br>**Regulator** — **Comment**<br>ERK — http://www.ncbi.nlm.nih.gov/pubmed/15239952<br>RSK2 is a well known ERK substrate in the cytoplasm and has been shown to undergo autophosphorylation after ERK phosphorylation.<br>PMID:15239952
SMAD	TGFBR	http://www.genenames.org/data/hgnc_data.php?hgnc_id=6768<br>http://www.genenames.org/data/hgnc_data.php?hgnc_id=6769<br>http://www.genenames.org/data/hgnc_data.php?hgnc_id=6770<br>http://www.ncbi.nlm.nih.gov/pubmed/21614932<br>SMAD2 = SMAD family member 2<br>SMAD3 = SMAD family member 3

		SMAD4 = SMAD family member 4<br>VARIABLE: SMAD gene (any isoform) expression level<br>Smad-dependent gene expression can provoke p38 activation in response to TGFbeta. GADD45beta was identified as the TGFbeta-inducible gene whose expression activates the p38 pathway.<br>PMID:21614932<br><br>**Regulator** / **Comment**<br>TGFBR: http://www.ncbi.nlm.nih.gov/pubmed/21614932<br>Smad-dependent gene expression can provoke p38 activation in response to TGFbeta. GADD45beta was identified as the TGFbeta-inducible gene whose expression activates the p38 pathway.<br>PMID:21614932
SOS	GRB2 & !RSK	http://www.genenames.org/data/hgnc_data.php?hgnc_id=11187<br>http://www.genenames.org/data/hgnc_data.php?hgnc_id=11188<br>http://www.ncbi.nlm.nih.gov/pubmed/17496910<br>http://www.ncbi.nlm.nih.gov/pubmed/18039929<br>http://www.ncbi.nlm.nih.gov/pubmed/21779497<br>http://www.ncbi.nlm.nih.gov/pubmed/9242373<br>SOS1 = son of sevenless homolog 1 (Drosophila)<br>SOS2 = son of sevenless homolog 2 (Drosophila)<br>VARIABLE: SOS (any isoform) recruitment by GRB2<br>Sos is recruited from the cytosol to the plasma membrane as a result of its constitutive interaction with Grb2. It is in an autoinhibited state.<br>PMID:17496910<br>The signaling pathways activated by FGFRs substantially overlap with those activated by EGFRs.<br>Grb2 molecules bound to FRS2 recruit the nucleotide exchange factor SOS, leading to the activation of the Ras-MAPK signaling cascade.<br>The process of activating Ras requires the recruitment of proteins responsible for initiating GDP/GTP exchange to the membrane, such as SOS (son of sevenless).<br>PMID:18039929<br>PI3K is a well characterised effector of RAS, through GRB2/SOS pathway.<br>PMID:21779497<br>p90 Rsk-2 is involved in SOS phosphorylation and may be important in down-regulation of the growth factor response.<br>PMID:9242373<br><br>**Regulator** / **Comment**<br>GRB2: http://www.ncbi.nlm.nih.gov/pubmed/17496910<br>Sos is recruited from the cytosol to the plasma membrane

		RSK	as a result of its constitutive interaction with Grb2. It is in an autoinhibited state.<br>PMID:17496910<br>http://www.ncbi.nlm.nih.gov/pubmed/9242373<br>p90 Rsk-2 is involved in SOS phosphorylation and may be important in down-regulation of the growth factor response.<br>PMID:9242373
SPRY	ERK	http://www.genenames.org/data/hgnc_data.php?hgnc_id=11270<br>http://www.ncbi.nlm.nih.gov/pubmed/15173823<br>SPRY2 = sprouty homolog 2 (Drosophila)<br>VARIABLE: SPRY2 phosphorylation level<br>Spry is induced by activated ERK, through phosphorylation on Tyr55. It positively regulates EGFR signalling by sequestering Cbl, whereas it negatively regulates FGFR signalling by sequestering Grb2 from FSR2.<br>PMID:15173823<br><br>**Regulator**<br>ERK	 <br><br><br><br><br><br><br>**Comment**<br>http://www.ncbi.nlm.nih.gov/pubmed/15173823<br>Spry is induced by activated ERK, through phosphorylation on Tyr55.<br>PMID:15173823
TAK1	TGFBR	http://www.genenames.org/data/hgnc_data.php?hgnc_id=6885<br>http://www.ncbi.nlm.nih.gov/pubmed/20060931<br>MAP3K7 = mitogen-activated protein kinase kinase kinase 7<br>VARIABLE: TAK1 phosphorylation level<br><br>Activated TGFBR1/TGFBR2 complex attracts the TAB2/TAB3 proteins which promote activation of TAK1 (by phosphorylation), leading to activation of p38 and JNK.<br>PMID:20060931<br><br>**Regulator**<br>TGFBR	 <br><br><br><br><br><br><br><br>**Comment**<br>http://www.ncbi.nlm.nih.gov/pubmed/20060931<br>Activated TGFBR1/TGFBR2 complex attracts the TAB2/TAB3 proteins which promote activation of TAK1 (by phosphorylation), leading to activation of p38 and JNK.<br>PMID:20060931
TAOK	ATM	http://www.genenames.org/data/hgnc_data.php?hgnc_id=29259<br>http://www.genenames.org/data/hgnc_data.php?hgnc_id=16835	

		http://www.genenames.org/data/hgnc_data.php?hgnc_id=18133<br>http://www.ncbi.nlm.nih.gov/pubmed/18855897<br>http://www.ncbi.nlm.nih.gov/pubmed/21614932<br>TAOK1 = TAO kinase 1<br>TAOK2 = TAO kinase 2<br>TAOK3 = TAO kinase 3<br>VARIABLE: TAOK (any isoform) phosphorylation level<br>Ataxia telangiectasia mutated (ATM) is activated in response to DNA damage and directly phosphorylates TAOK.<br>PMID:18855897<br>TAO kinases are MAP3Ks that function upstream of p38 and JNK.<br>PMID:21614932<br><br>**Regulator** — **Comment**<br>ATM — http://www.ncbi.nlm.nih.gov/pubmed/18855897<br>Ataxia telangiectasia mutated (ATM) is activated in response to DNA damage and directly phosphorylates TAOK.<br>PMID:18855897
TGFBR	TGFBR_stimulus	http://www.genenames.org/data/hgnc_data.php?hgnc_id=11772<br>http://www.genenames.org/data/hgnc_data.php?hgnc_id=11773<br>http://www.genenames.org/data/hgnc_data.php?hgnc_id=11774<br>http://www.ncbi.nlm.nih.gov/pubmed/21614932<br>http://www.ncbi.nlm.nih.gov/pubmed/17673906<br>TGFBR1 = transforming growth factor, beta receptor 1<br>TGFBR2 = transforming growth factor, beta receptor II (70/80kDa)<br>TGFBR3 = transforming growth factor, beta receptor III<br>VARIABLE: TGFBR activation level<br>Smad-dependent gene expression can provoke p38 activation in response to TGFbeta.<br>PMID:21614932<br>TGF-beta-induced ShcA phosphorylation induces ShcA association with Grb2 and Sos, thereby initiating the well-characterised pathway linking receptor tyrosine kinases with Erk MAP kinases.<br>PMID:17673906
TGFBR_stimulus	input	Model input<br>VARIABLE: any stimulus able to induce TGFBR activation
