# Supplementary material for: Integrative Modelling of the Influence of MAPK Network on Cancer Cell Fate Decision
Source: PLoS Comput Biol. 2013 Oct 24;9(10):e1003286. doi: 10.1371/journal.pcbi.1003286 (PMC3821540; doi:10.1371/journal.pcbi.1003286)
Supplement: Text S1 — Supplementary text encompassing two sections. The first one describes how we derived the logical model from the reaction map. The second one demonstrates how we checked that all cyclic attractors obtained for the MAPK model reductions indeed correspond to attractors of the original comprehensive model. (PDF) [file pcbi.1003286.s009.pdf]

Text S1

1. From the reaction map to the logical model

As mentioned in the main text, the logical model was manually built starting from the information stored in the reaction map. In this paragraph, we describe in more details how this process was carried out.

1.1 Definition of the specific biological question

We built the reaction map with the aim to generically represent the influence of the MAPK network on cell fate decision. Subsequently, we defined a specific biological question of interest, *i.e.* studying the dynamics of such events in response to a subset of stimuli. We considered two particular receptors of the RTK family, namely EGFR and FGFR3, which have been shown to determine different behaviours in different subtypes of urinary bladder cancer. Thus, our goal was to recapitulate such behaviours and get insights into the underlying mechanisms through a logical model.

1.2 Construction of the regulatory graph

To address our biological questions, we generated the regulatory graph depicted in Figure 2.

First of all, we selected the inputs (*i.e.* the nodes from which modelled signals are supposed to arise) and the readouts (observed phenotypes) of the graph. We defined EGFR_stimulus and FGFR3_stimulus to represent any kind of event able to activate the corresponding receptors (e.g. EGFR_stimulus can be associated with EGF signals). Similarly, we defined TGFBR_stimulus and DNA_damage as inputs, that were introduced in order to represent major anti-proliferative stimuli acting on MAPK network, counteracting the mainly proliferative effects of growth factors. Please note that the additional stimuli reported in the reaction map, such as GPCR, TNFRs, IL1R (which are also strongly related with MAPK network-dependent cell fate decision), will be gradually considered for future developments of our model. Our readouts were Apoptosis, Proliferation and Growth_Arrest. Additionally, we included the three MAPKs (ERK, p38, JNK) in the graph.

Starting from this initial scheme (cf. Figure 2), we gradually added components and interactions based on the reactions reported in the reaction map. In particular, from the

stimuli, we examined all the downstream reactions leading to the phenotypes, and represented the flow of information in a more abstract way, by using positive and negative influences between selected key components. Here, we give some examples of how we modelled the pathways generated by EGFR and FGFR3 stimuli.

Activated EGFR and FGFR3 both correspond to RTK component in the CellDesigner map. Thus, their downstream effects mainly overlap. For instance, RTKs can trigger MAPK cascades through GRB2. In particular, EGFR can directly recruit the complex GRB2/SOS (re25), whereas the intermediate action of FRS2 is needed following FGFR3 stimulus (re273 and re25). In Figure 2, this corresponds to EGFR → GRB2 → SOS and to FGFR3 → FRS2 → GRB2 → SOS, respectively. In turn, the association of GRB2 and FRS2 results in the degradation of FRS2 and FGFR3 (re273), that we modelled through negative influences from GRB2 towards FRS2 and FGFR3. Once SOS is recruited, it can activate RAS (re9), which is in turn able to trigger the ERK cascade (RAF/MEK/ERK) in several sub-cellular compartments (re13, re19, re102, and downstream). RAS can also activate p38 and JNK cascades (re137, re140, and downstream). These aspects were modelled by positive influences from RAS to RAF and to MAP3K1_3, the latter positively influencing p38 and JNK (Figure 2). Note that we do not assume mutual inhibition between components that compete for acting on the same species.

In some cases, we compressed linear pathways by reporting only a few key components. For instance, despite PLCγ activation follows SRC recruitment by RTKs, we did not explicitly represented SRC in the regulatory graph. This was possible as no other events are associated with SRC in the map. For similar reasons, Ca2+ is not present in the regulatory graph, as well.

Furthermore, in the case of ERK signalling, as mentioned in the main text, we did not consider all the possible effects of different sub-cellular localisations or scaffold proteins (in order to keep the same level of detail with p38 and JNK cascades). This led us to compact several pathways depicted in the reaction map into a unique core ERK cascade in the regulatory graph. It will be possible to further detail this aspects when similar information concerning p38 and JNK pathways will be available.

1.3 Assignment of logical rules

The regulatory graph can be considered as part of the logical model, as its nodes represent the model variables, and its arcs represent the influences on the basis of which we can build the logical rules associated with each variable.

In our case, we decided to keep all the variables Boolean. Thus, the logical rule associated with a node (*i.e.* variable) is defined by a combination of presences (level 1) or absences (level 0) of all its regulators,using the Boolean operators AND, OR and NOT. The rest of this section describe how we proceed to define the logical rules.

Let us suppose a set of nodes $S=\{s_1,...,s_N\}$ acts upon the node *t*. In general, we assumed that:

- When all the nodes of *S* exert a positive (negative) influence on *t* such that the high level of at least one of them is sufficient to activate (inhibit) *t*, the operator OR was used. We assumed this unless evidence suggested that a (sub-)set of components needed to be present at the same time. This is the case, for example, of BCL2 (Figure 2), for which the presence of both CREB (determining its expression) and PI3K (determining its activation) is necessary to determine its activation: an AND was then used in such case (cf. supplementary Table S2).

- When the nodes of *S* exert contrasting influences on *t*, our basic assumption was that one inhibition is sufficient to disrupt any number of concurrent activations. For example, EGFR node receives positive influences from EGFR_stimulus and SPRY, and negative influences from PKC and GRB2: then, when at least one between PKC and GRB2 is present, EGFR will be pushed to 0; otherwise, in presence of either EGFR_stimulus or SPRY, EGFR will be pushed to 1. However, several exceptions arose during model simulations, leading us to tune some rules in order to let the model fit the published experimental data. For example, this is the case of p53, for which we needed to “weaken” the inhibitory role of MDM2: when both p38 and ATM are present, the presence of MDM2 is not sufficient to inhibit p53; whereas, when at most one between p38 and ATM is present, the presence of MDM2 is sufficient to inhibit p53 (cf. Table S2).

2 All cyclic attractors of the MAPK reduced models reflect cyclic attractors of the original model

Our model reduction algorithm guarantees the preservation of all attractors (cf. Naldi et al, 2011). In particular, the stable states are all preserved, and each stable state of the reduced model is a projection (on the reduced state space) of a stable state of the original model. All the cyclic attractors are also preserved, but the generation of novel, potentially "spurious" cyclic attractors is not excluded when reducing a logical model. This can correspond to the isolation of some transient strongly connected components in the state transition graph due to compression of dynamics.

Thus, as we performed our simulations on reduced model versions, we needed to check whether the cyclic attractors observed correspond to cyclic attractors of the original large model.

For instance, let us consider the simulation r4, where we set up FGFR3 activating mutation and all inputs set to 0 as configuration. We obtained two stable states and one cyclic attractor in the red1 model version (cf. supplementary Dataset S3 - r4). We wanted to check whether the cyclic attractor was not spurious. In other words, starting from any state belonging to this attractor, we should obtain a unique strongly connected component (and thus a cyclic attractor) as state transition graph of the large model. In order to reduce the computational cost of such simulation, we can set as initial states the values for the stable variables obtained in the attractor of the reduced model. Furthermore, we can derive the values of the other (hidden) stable variables in the attractor based on the logical rules. For example, the considered cyclic attractor in the reduced model is characterised by FGFR3 stably set to 1 (cf. supplementary Dataset S3 - r4). We can thus derive the corresponding value for the hidden variable PLCG in the large model, which is dependent on FGFR3 (PI3K = FGFR3 | EGFR, cf. supplementary Table S2), and iteratively derive the stable value of (most of) the other non-oscillating hidden variables. This allows us to reduce the computational cost of the simulation on the large model. In this specific example, we could retrieve the stable value of all variables but 3 (GRB2, FRS2, SOS). Thus, we performed a simulation on the large model with FGFR3 activating mutation and all inputs set to 0. The set of initial states was formed by 8 states, that is all the possible combinations of the three oscillating variables, with all the other variables set to the stable value retrieved (cf. re33 in supplementary Dataset S4 – GINsim file corresponding to the large model).

We performed such analysis for all the identified attractors listed in supplementary Dataset S3, which all resulted non-spurious.

References

Naldi A, Remy E, Thieffry D, Chaouiya C (2011) Dynamically consistent reduction of logical regulatory graphs. *Theoretical Computer Science* **412**: 2207–2218.
