# Supplementary material for: Integrative Modelling of the Influence of MAPK Network on Cancer Cell Fate Decision
Source: PLoS Comput Biol. 2013 Oct 24;9(10):e1003286. doi: 10.1371/journal.pcbi.1003286 (PMC3821540; doi:10.1371/journal.pcbi.1003286)
Supplement: Text S2 — Hierarchical transition graphs associated with receptor alterations. Model dynamics following either EGFR over-expression or FGFR3 activating mutation (with all inputs set to 0, throughout the simulations) are depicted in two separated graphs, which were obtained using the reduced model version red1. For sake of simplicity, simulations were performed by using a single initial state with all the remaining variables set to 0 (the salient dynamics were preserved in these cases – cf. Dataset S3). The resulting hierarchical transition graphs (see Methods) are composed by different classes of nodes, emphasising strongly connected components (blue), and linear (non circular) pathways (pink). The attractors reached are represented at the bottom of the figures. Attractor colours refer to the corresponding phenotypes: red for proliferation, green for apoptosis, grey for no decision. Stables states are denoted by rectangles, while cyclic attractors are denoted by circles. The accompanying tables give the composition of each node of the corresponding HTG. For instance, the node cc1 of the HTG obtained for FGFR3 activating mutation corresponds to a strongly connected component of the state transition graph. The number of states belonging to it (i.e. 24), as well as the list of these states are listed in the table (asterisks denote all possible values for the corresponding variable). (PDF) [file pcbi.1003286.s010.pdf]

Text S2

Hierarchical transition graphs associated with receptor alterations

Model dynamics following either EGFR over-expression or FGFR3 activating mutation are depicted in two separated graphs, which were obtained using the reduced model version red1. For sake of simplicity, simulations were performed by using a single initial state with all the remaining variables set to 0 (the salient dynamics were preserved in these cases – cf. Dataset S3). The resulting hierarchical transition graphs (see Methods) are composed by different classes of nodes, emphasising strongly connected components (blue), and linear (non circular) pathways (pink). The attractors reached are represented at the bottom of the figures. Attractor colours refer to the corresponding phenotypes: red for proliferation, green for apoptosis, grey for no decision. Stables states are denoted by rectangles, while cyclic attractors are denoted by circles. The accompanying tables give the composition of each node of the corresponding HTG. For instance, the node cc1 of the HTG obtained for FGFR3 activating mutation corresponds to a strongly connected component of the state transition graph encompassing 24 states. These states are listed in a compact form, where asterisks denote all possible values (O or 1) for the corresponding variable (the two rows for cc1 thus represent 24 states).

1. EGFR over-expression

1.1 HTG obtained with EGFR over-expression

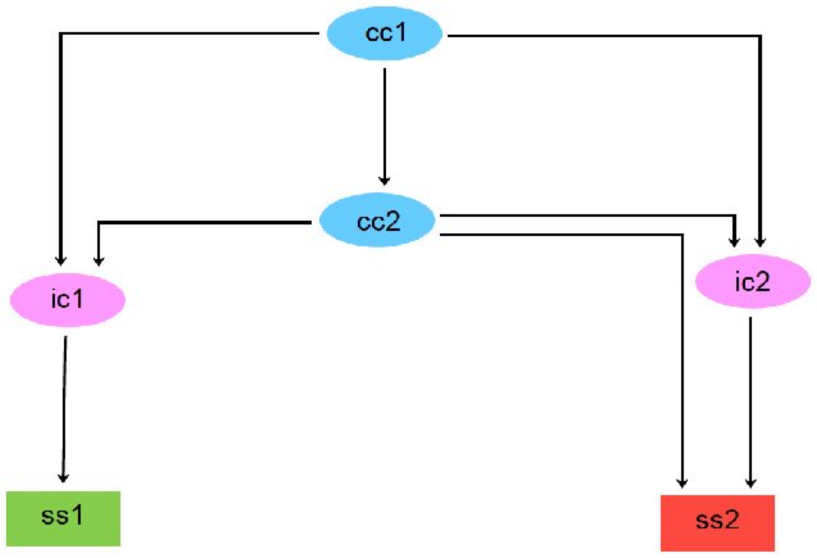

1.2 Composition of the HTG states associated with EGFR over-expression

Node ID	Number of states	Apoptosis	Growth_Arrest	Proliferation	ERK	p53	EGFR	FGFR3	FRS2	PI3K	AKT	MSK	p14	PTEN
ct1	32	*	*	0	0	*	1	0	0	0	0	*	0	*
ct2	112	*	*	0	0	0	1	0	0	1	*	*	*	*
		*	*	0	0	1	1	0	0	1	*	0	*	*
		*	*	0	0	1	1	0	0	1	*	1	0	*
ic1	71	0	0	0	1	0	1	0	0	0	0	1	0	*
		0	0	0	1	0	1	0	0	1	*	1	*	*
		0	0	1	1	0	1	0	0	1	0	1	*	*
		0	0	1	1	0	1	0	0	1	1	1	0	*
		0	0	1	1	0	1	0	0	1	1	1	1	1
		0	1	0	1	0	1	0	0	0	0	1	0	*
		0	1	0	1	0	1	0	0	1	*	1	*	*
		0	1	1	1	0	1	0	0	1	*	1	*	*
		1	*	0	1	0	1	0	0	0	0	1	0	*
		1	*	0	1	0	1	0	0	1	*	1	*	*
		1	*	1	1	0	1	0	0	1	*	1	*	*
ic2	23	0	*	0	0	1	1	0	0	0	0	1	1	*
		0	*	0	0	1	1	0	0	1	*	1	1	*
		1	0	0	0	1	1	0	0	0	0	1	1	*
		1	0	0	0	1	1	0	0	1	*	1	1	*
		1	1	0	0	1	1	0	0	0	0	1	1	*
		1	1	0	0	1	1	0	0	1	0	1	1	0
		1	1	0	0	1	1	0	0	1	1	1	1	*
ss1	1	0	0	1	1	0	1	0	0	1	1	1	1	0
ss2	1	1	1	0	0	1	1	0	0	1	0	1	1	1

2. FGFR3 activating mutation

2.1 HTG obtained with FGFR3 activating mutation

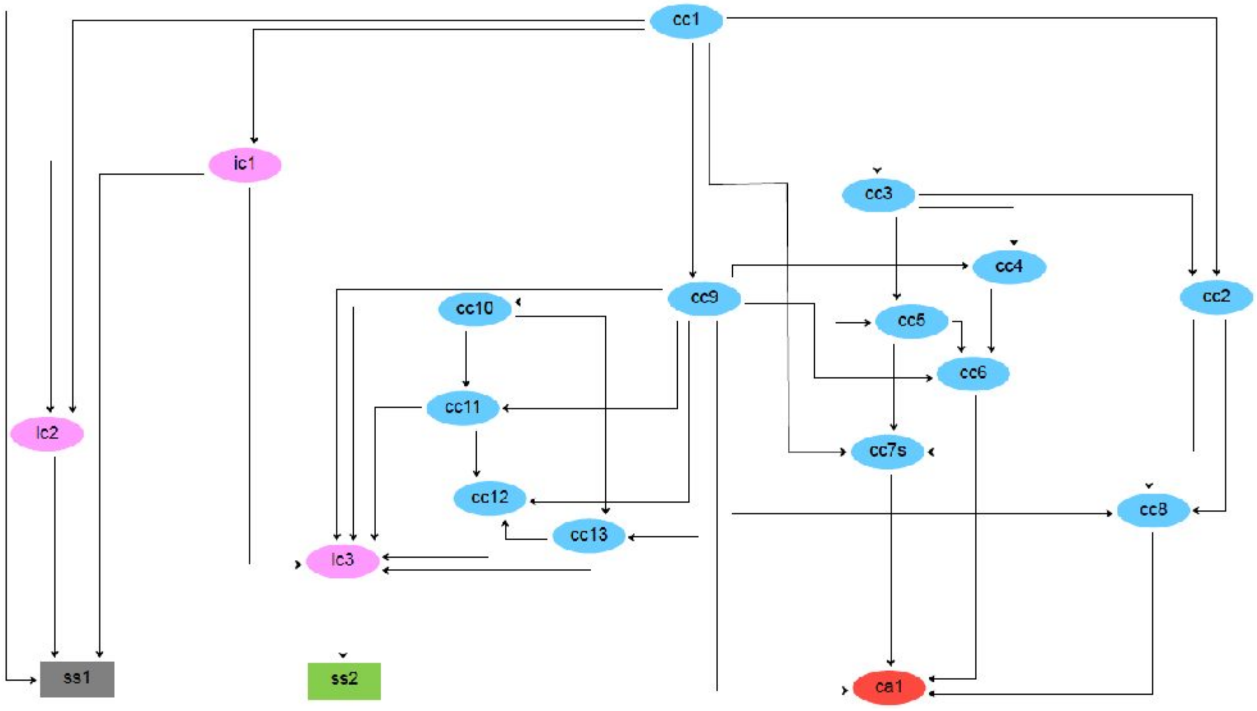

2.2 Composition of the HTG states associated with FGFR3 activating mutation

Node ID	Number of states	Apoptosis	Growth_Arrest	Proliferation	ERK	p53	EGFR	FGFR3	FRS2	PI3K	AKT	MSK
cc1	24	*	*	0	0	0	0	1	*	0	0	*
		*	*	0	0	1	0	1	*	0	0	0
cc2	2	0	1	0	0	1	0	1	*	0	0	1
cc3	2	0	0	0	0	1	0	1	*	0	0	1
cc4	2	0	0	0	0	1	0	1	*	1	0	1
cc5	2	1	0	0	0	1	0	1	*	0	0	1
cc6	2	1	0	0	0	1	0	1	*	1	0	1
cc7	2	1	1	0	0	1	0	1	*	0	0	1
cc8	2	0	1	0	0	1	0	1	*	1	0	1
cc9	24	*	*	0	0	0	0	1	*	1	0	*
cc10	4	1	1	0	0	0	0	1	*	1	1	*
cc11	4	0	1	0	0	0	0	1	*	1	1	*
cc12	4	0	0	0	0	0	0	1	*	1	1	*
cc13	4	1	0	0	0	0	0	1	*	1	1	*
ic1	4	*	*	0	1	0	0	1	1	0	0	1
ic2	3	0	1	0	1	0	0	1	0	0	0	1
		1	*	0	1	0	0	1	0	0	0	1
ic3	23	0	0	0	1	0	0	1	*	1	*	1
		0	0	1	1	0	0	1	1	1	1	1
		0	1	0	1	0	0	1	*	1	*	1
		0	1	1	1	0	0	1	*	1	1	1
		1	*	0	1	0	0	1	*	1	*	1
		1	*	1	1	0	0	1	*	1	1	1
ss1	1	0	0	0	1	0	0	1	0	0	0	1
ss2	1	0	0	1	1	0	0	1	0	1	1	1

NB: we further reduced “red1” model version, by hiding p14 and PTEN, in order to further reduce the dimension of the HTG associated with this simulation. Results are coherent with the ones obtained with “red1” model (cf. supplementary Dataset S3 - r4).
